# In vitro biomimetic platforms featuring a perfusion system and 3D spheroid culture promote the construction of tissue-engineered corneal endothelial layers

**DOI:** 10.1038/s41598-017-00914-1

**Published:** 2017-04-10

**Authors:** Shanyi Li, Yuting Han, Hao Lei, Yingxin Zeng, Zekai Cui, Qiaolang Zeng, Deliang Zhu, Ruiling Lian, Jun Zhang, Zhe Chen, Jiansu Chen

**Affiliations:** 1 Key Laboratory for Regenerative Medicine, Ministry of Education, Jinan University, Guangzhou, 510632 P.R. China; 2 Institute of Ophthalmology, Medical College, Jinan University, Jinan University, Guangzhou, 510632 P.R. China; 3 The Department of Ophthalmology, the First Clinical Medical College, Jinan University, Guangzhou, 510632 P.R. China; 4 Key Laboratory of Optoelectronic Information and Sensing Technologies of Guangdong Higher Educational Institutes, Jinan University, Guangzhou, 510632 P.R. China; 5 Aier Eye Institute, #198 Furong Middle Road, Changsha, 410015 P.R. China; 6 Department of Applied Physics, South China Agricultural University, Guangzhou, 510632 P.R. China

## Abstract

Corneal endothelial cells (CECs) are very important for the maintenance of corneal transparency. However, *in vitro*, CECs display limited proliferation and loss of phenotype via endothelial to mesenchymal transformation (EMT) and cellular senescence. In this study, we demonstrate that continuous supplementary nutrition using a perfusion culture bioreactor and three-dimensional (3D) spheroid culture can be used to improve CEC expansion in culture and to construct a tissue-engineered CEC layer. Compared with static culture, perfusion-derived CECs exhibited an increased proliferative ability as well as formed close cell-cell contact junctions and numerous surface microvilli. We also demonstrated that the CEC spheroid culture significantly down-regulated gene expression of the proliferation marker *Ki67* and EMT-related markers *Vimentin* and *α*-*SMA*, whereas the gene expression level of the CEC marker *ATP1A1* was significantly up-regulated. Furthermore, use of the perfusion system in conjunction with a spheroid culture on decellularized corneal scaffolds and collagen sheets promoted the generation of CEC monolayers as well as neo-synthesized ECM formation. This study also confirmed that a CEC spheroid culture on a curved collagen sheet with controlled physiological intraocular pressure could generate a CEC monolayer. Thus, our results show that the use of a perfusion system and 3D spheroid culture can promote CEC expansion and the construction of tissue-engineered corneal endothelial layers *in vitro*.

## Introduction

Corneal endothelial cells (CECs), which reside at the inner surface of the cornea, maintain corneal transparency by regulating stromal hydration via their barrier and pump functions^1^. When the cell density of CECs falls below the critical level required to maintain normal corneal hydration, corneal edema usually occurs and vision is gradually impaired, thereby requiring corneal transplantation to restore normal vision^1^. Recently, there has been enormous interest in strategies involving regeneration of the corneal endothelium as an efficient alternative to endothelial keratoplasty, in which only the posterior host corneal layers are replaced by a suitable endothelial graft to restore endothelial function^2–4^. Tissue-engineered endothelial grafts that utilize biomaterials and biomimetic scaffolds and incorporate the patient’s own cells have become a prospective alternative to allografts in the treatment of endothelial defects^5^. Although several studies have investigated the use of amniotic membranes and silk fibroin membranes as corneal substitutes, there are still insufficient data to permit further clinical trials^3, 6, 7^. The challenge of producing CEC layers or cells for endothelial cell therapy by tissue engineering is the phenotypic instability of CECs. Therefore, solving this problem will require the use of versatile cell culture technology and materials to produce cells that can serve as artificial endothelial grafts.

CECs retain the ability to proliferate *in vitro* under the proper culture conditions^8^. However, after long-term subculture, they tend to display limited proliferation, senescence or fibroblastic transformation with morphological, physiological and functional loss caused by dedifferentiation^1, 9–11^. Hence, there is a need to develop culture systems for CECs *in vitro* that not only promote proliferation but also counter dedifferentiation events such as the endothelial-to-mesenchymal transition (EndoMT or EMT)^12^. The cellular microenvironment, as one of the primary determining factors of cellular activity in the human body, impacts cell morphology and physiological functions^13, 14^. Accumulating evidence has suggested that mechanical factors play an important role in influencing cell growth, structure, and function^15, 16^. Flow perfusion systems provide a flexible platform for developing a controllable biomimetic environment that can be adapted for use in various investigations of dynamic cell culture under conditions of rheological stress and hydrostatic pressure^17–19^. Flow perfusion systems such as bioreactors enable the continuous and constant supply of nutrients and oxygen to cells and can be used to improve the environment for *in vitro* tissue-engineered conditions^18, 20^. Therefore, flow perfusion systems may be an efficient method for the construction of CEC layers and the observation of morphological and physiological changes in CECs. Mannis *et al*. studied the morphology of CECs under *in vivo* anterior chamber perfusion. Clinically, they were unable to detect any signs of gross corneal dysfunction with hypothermic perfusion at a flow rate of 5 ml/min^21^. Brunette *et al*. maintained clear human corneas using incubation and perfusion for 3 weeks. The endothelial cells remained viable and functional^22^. Thiel *et al*. reported that a chamber device was a reliable tool for *in vitro* drug penetration and toxicity studies in isolated perfused corneoscleral tissue^23^. However, to our knowledge, there are few or no published reports concerning perfusion culture for the construction of tissue-engineered CEC layers *in vitro*. To improve the environment for cells and developing specialized tissues, the MINUSHEET perfusion culture system was developed in 1990^24^. In this system, the pigmented epithelium and neighbouring neurons of the intact retina maintain a perfect morphology for a culture period of at least 10 days. The development of a gingival epithelium or co-culture of keratinocytes and osteoblast-like cells in a perfusion container yields much better results than those obtained under static culture conditions. The MINUSHEET perfusion culture system thus is advantageous for use in the engineering of connective tissue, the generation of nervous tissue, and the development of muscle tissue^18^.

Another type of cell culture, spheroid (SP) culture, which mimics the microenvironment *in vivo* to yield a multicellular mass mediated by cadherin, exhibits several advantages over conventional two-dimensional (2D) culture^25, 26^. The precursors obtained from the CEC spheroids possess longer telomeres and higher telomerase activity^27^, and CEC-derived spheroid therapy has been used in a rabbit CEC deficiency model^28^. Our previous studies also found that CEC spheroids treated with Y-27632 are injectable *in vitro* and have important implications for the favourable treatment of CEC deficiency^29^.

The available matrices for CEC sheet generation have progressed from natural membranes, biological polymers, and biosynthetic materials to completely synthetic materials^30^. Acellular scaffolds such as the amniotic membrane and corneal stroma transmit light and possess mechanical properties that may support CEC attachment for transplantation applications *in vivo* ^6, 31^. However, there is currently no reliable, standardized decellularization protocol to remove immunoreactive material and at the same time maintain tissue properties such as corneal curvature^32, 33^. Our previous report showed that short-term chemical-frozen decellularization of the bovine stromal matrix allowed keratocytes to maintain their dendritic shape, reticular arrangement and phenotypic stability even in the presence of 10% FBS. Collagen is the most abundant protein in the cornea and is the primary structural component of corneal tissue^34^. Recently, researchers have demonstrated that collagen vitrigel with a spherical curve, such as porcine-derived atelocollagen vitrigel, can be used as a spherical biomimetic scaffold for artificial endothelial grafts^33, 35^.

In this study, we for the first time employed the MINUSHEET perfusion system to expand bovine CECs and construct CEC layers *in vitro*. Furthermore, we describe the use of a spheroid process using agarose micromolds and provide insight into how bovine CEC functions are improved in spheroid culture. Bovine CECs were used because that made it possible to obtain 25–50 age-matched 1-2-year-old eyes at one time and thereby to obtain the large number of cells required for perfusion and spheroid studies, which was not possible using human tissue^36^. Cultured bovine CECs provided us with an excellent *in vitro* model for the study of the differentiated functions of the corneal endothelium^37^. The investigation of cellular behaviour in the bovine CEC model can also be revealing for human CEC biology and applications. To demonstrate this system’s application in tissue-engineered CEC construction, conventional decellularized bovine corneal scaffolds and flat or spherically curved collagen sheets were investigated. Our objective was to explore whether the use of biomimetic platforms involving a perfusion system and three-dimensional (3D) spheroid culture not only promote CEC proliferation and counter EMT but also enhance the construction of tissue-engineered corneal endothelial layers close to the native cornea.

## Results

### Enhancement of the oxygen supply and CEC proliferation in the perfusion system

**Figure 1:**
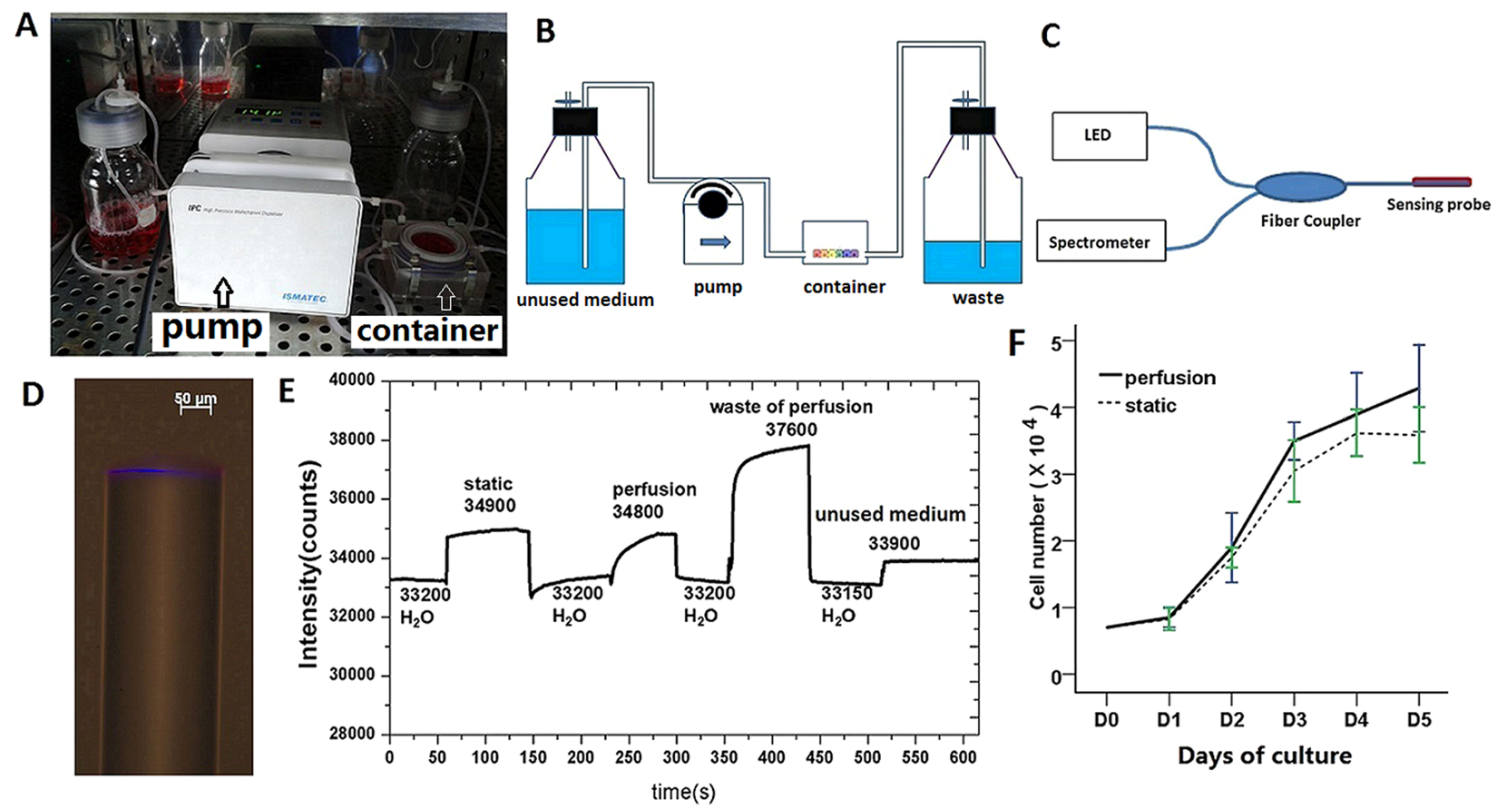
Enhancement of the oxygen supply and CEC proliferation in the perfusion system. (**A**) Photograph of the perfusion system in a 37 °C incubator containing 5% CO_2_. (**B**) Photographic illustration of the perfusion system. (**C**) Schematic of the sensor system. (**D**) Photograph of the microsensor. (**E**) Intensity values of the microsensor for dissolved oxygen. (**F**) Growth curves of CECs in the static and perfusion systems after seeding at an initial cell number of 0.7 × 10^4^ cells/well.

The measurement from the optical fibre oxygen fluorescence microsensor showed that the relative oxygen intensity in the waste medium of the perfusion was the strongest, followed by that in the medium of the static culture and then that in the perfusion medium. The value in the unused medium was the weakest (Fig. 1E). Figure 1E also shows that the relative intensities in water (H_2_O) were maintained at nearly the same value, indicating that the microsensor remained stable for the duration of the measurement. The dissolved oxygen concentration is inversely proportional to the luminescence intensity. Thus, the dissolved oxygen concentration is the highest in the unused medium, followed by the perfusion medium, the medium from cells grown under static conditions and the waste medium.

**Figure 2:**
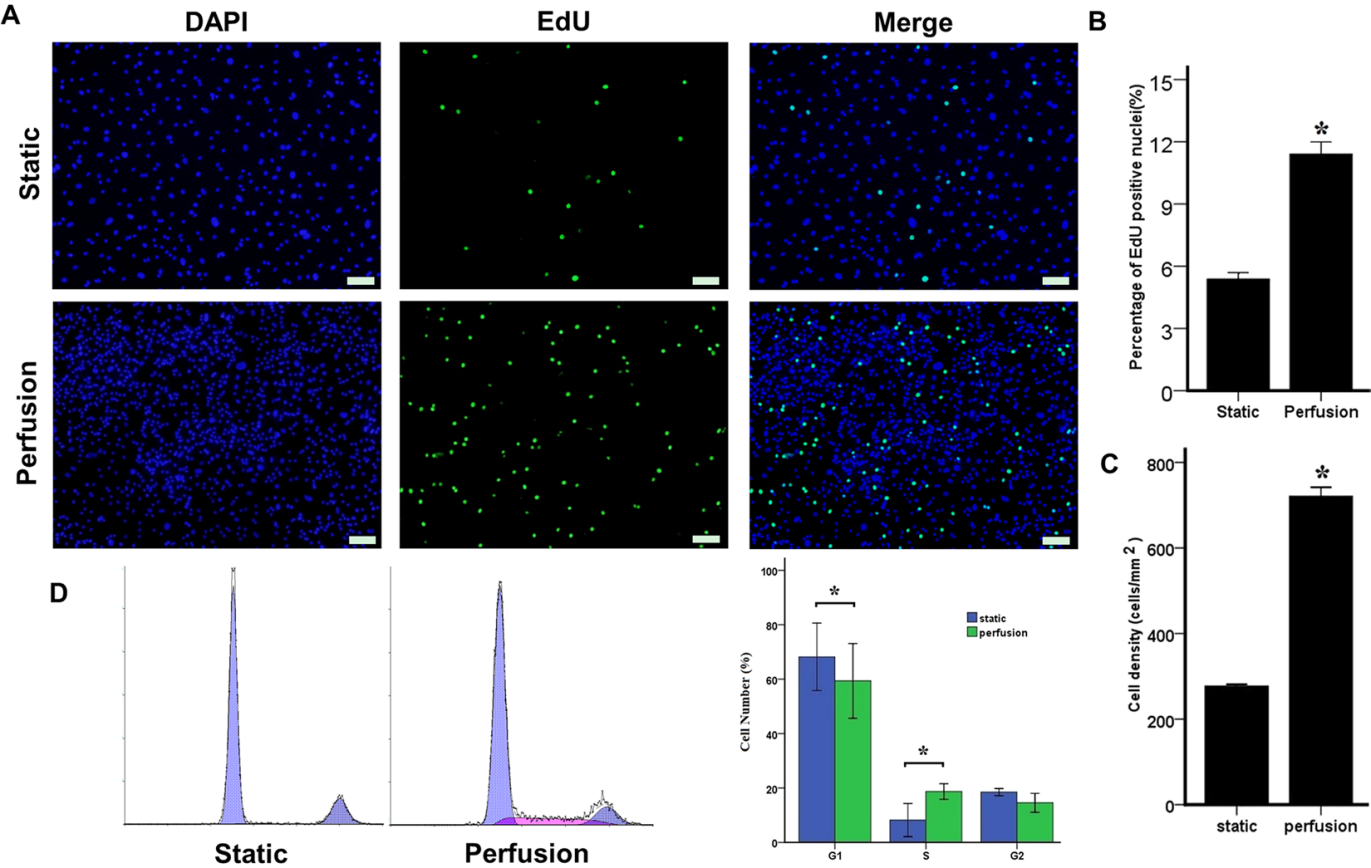
Effects of the perfusion system on the growth ability of CECs. (**A**) EdU assay of CECs in the static and perfusion systems. Scale bar: 100 µm. (**B**) The proliferation of CECs was evaluated based on the ratio of EdU-positive cells to total cells. (**C**) Quantification of the cell density in the static and perfusion systems. (**D**) Flow cytometry analysis of the cell cycle in the static and perfusion systems. The data are presented as the mean ± SD of three independent experiments. Differences with *P < 0.05 were considered to be statistically significant.

Compared with static-derived CECs, perfusion-derived CEC cultures contained greater numbers of cells at later times (Fig. 1F). To investigate the effect of the perfusion system on the proliferation of CECs, the number of EdU-positive cells and the cell cycle were also analysed. Cells cultured in the perfusion system displayed more EdU-positive cells than those in the static system on day 3 (D3) (Fig. 2A). The percentage of EdU-positive CEC nuclei in cultures grown in the static system and in the perfusion system on D3 were 5.38 ± 0.59% and 11.41 ± 1.0%, respectively (Fig. 2B). There was a significant increase in the cell density in the perfusion system (721 ± 36 cells/mm^2^) compared with that in the static condition (277 ± 8 cells/mm^2^) (Fig. 2C) after 5 days of culture. Similarly, PI flow cytometry demonstrated that the cell cycle distribution of CECs in the perfusion system was obviously promoted. The percentages of cells in the G1 and S phases on D3 in the perfusion system were 66.66 ± 6.90% and 17.55 ± 1.47%, respectively, whereas those in the static condition were 75.93 ± 6.70% and 6.98 ± 4.37%, respectively (Fig. 2D). These results demonstrate that perfusion culture with continual supplement nutrition supports greater CEC proliferation during expansion than the static culture.

### Maintenance of the CEC phenotype in the perfusion system

**Figure 3:**
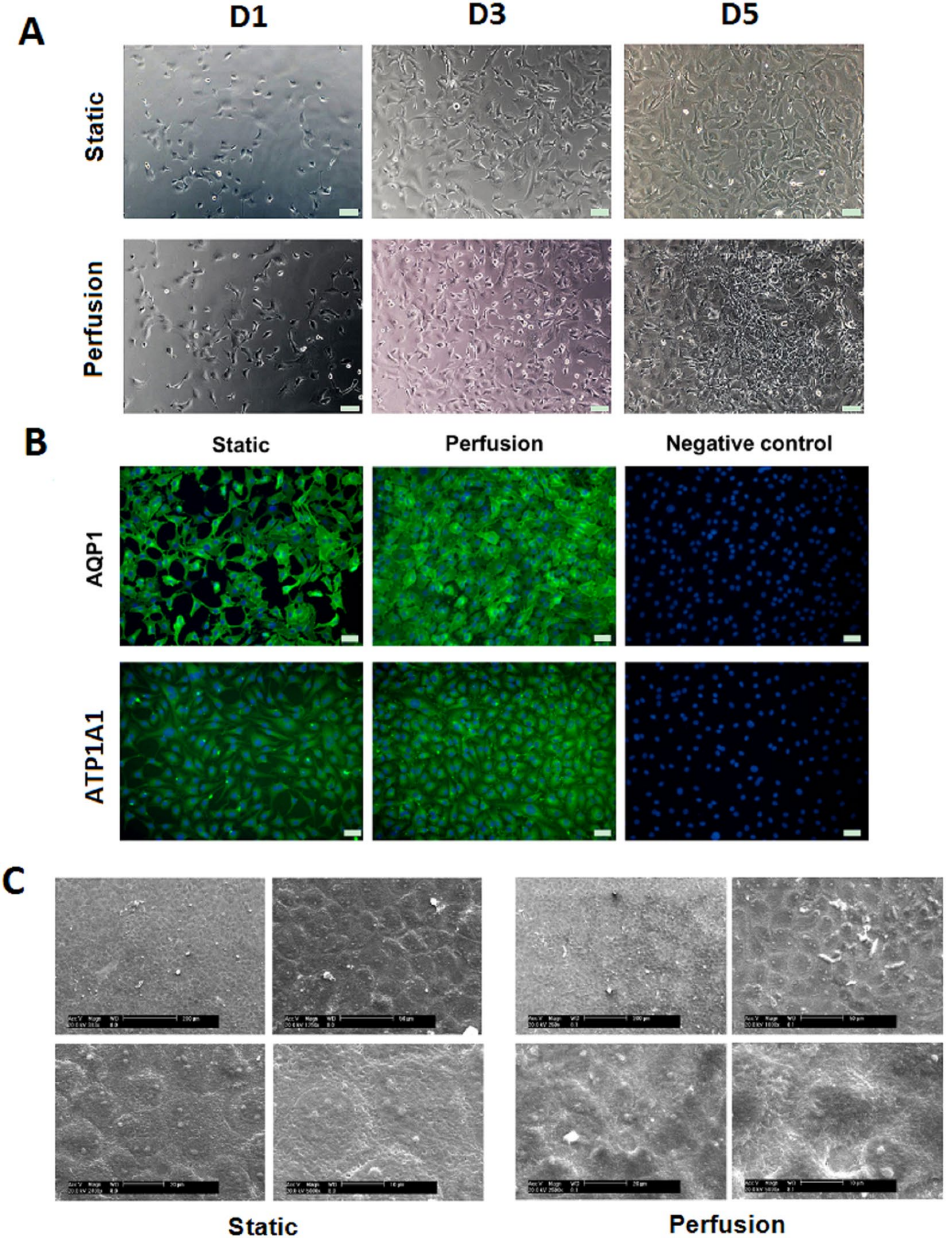
Maintenance of the CEC phenotype in the perfusion system. (**A**) Representative phase-contrast images of CECs in the static and perfusion systems at D1, D3 and D5. Scale bar: 100 μm. (**B**) Immunofluorescence images of AQP1 and ATP1A1 staining. Scale bar: 50 μm. (**C**) SEM images of CECs on glass carriers in the static and perfusion systems.

We further explored whether the CEC phenotypes were maintained in the perfusion system after enhancement of the oxygen supply, and CEC proliferation in such a dynamic culture was found. Bovine CECs cultured in the static system or the perfusion system on days 1 (D1), 3 (D3) and 5 (D5) were imaged under an inverted contrast microscope. CECs cultured in the static system exhibited polygonal morphology. Compared with cells in static culture, CECs grown in the perfusion system on D5 were characterized by polygonal, especially hexagonal, morphology, smaller size, and higher cell density (Fig. 3A). Immunofluorescence staining revealed that the protein expression levels of AQP1 and ATP1A1 in CECs in both the static and perfusion systems were positive (Fig. 3B). CECs passaged on 2D culture TCPS after static or perfusion culture expressed positive Calcein-AM live cell staining and the CEC phenotypic markers AQP1, vimentin and N-cadherin (Fig. S2). SEM micrographs showed that CECs in static culture were arranged in flat monolayers with microvilli randomly distributed over the smooth cell surface, whereas the CEC monolayer in the perfusion system was characterized by a raised and rough surface topography with more microvilli (Fig. 3C). These results suggest that CEC phenotype and surface topography are maintained under perfusion culture conditions.

### Impediment of EMT in CECs by biomimetic platforms of CEC spheroids and perfusion culture

We first investigated the characterization of CEC spheroids in static culture. CECs were cultured in multiwell agarose micromolds. Preliminary spheroids formed on day 1 after seeding. The cell viability assay (Live/Dead assay) demonstrated that most of the cells in the CEC spheroids were viable during seven days of culture, showing an intense green fluorescence in the live cytoplasm from Calcein AM staining. Dead cells were located mainly in the centres of CEC spheroids after five days of culture; red fluorescence was apparent in dead cell nuclei from the EthD-III staining (Fig. S3). Live/Dead staining also revealed that the spheroid diameters were approximately 140 μm (small spheroids), 300 μm (middle spheroids) and 600 μm (large spheroids) after 600, 3,000 and 27,000 cells per microwell were seeded and cultured for 3 days, respectively (Fig. S4). QPCR revealed significant up-regulation of the endothelial markers *ATP1A1*, *AQP1 and N*-*cadherin*, the proliferation marker *Ki67* and the mesenchymal markers *Vimentin* and *α*-*SMA*, particularly in small spheroids (Fig. S4D). Hence, small CEC spheroids cultured for 3 days were chosen for subsequent experiments.

**Figure 4:**
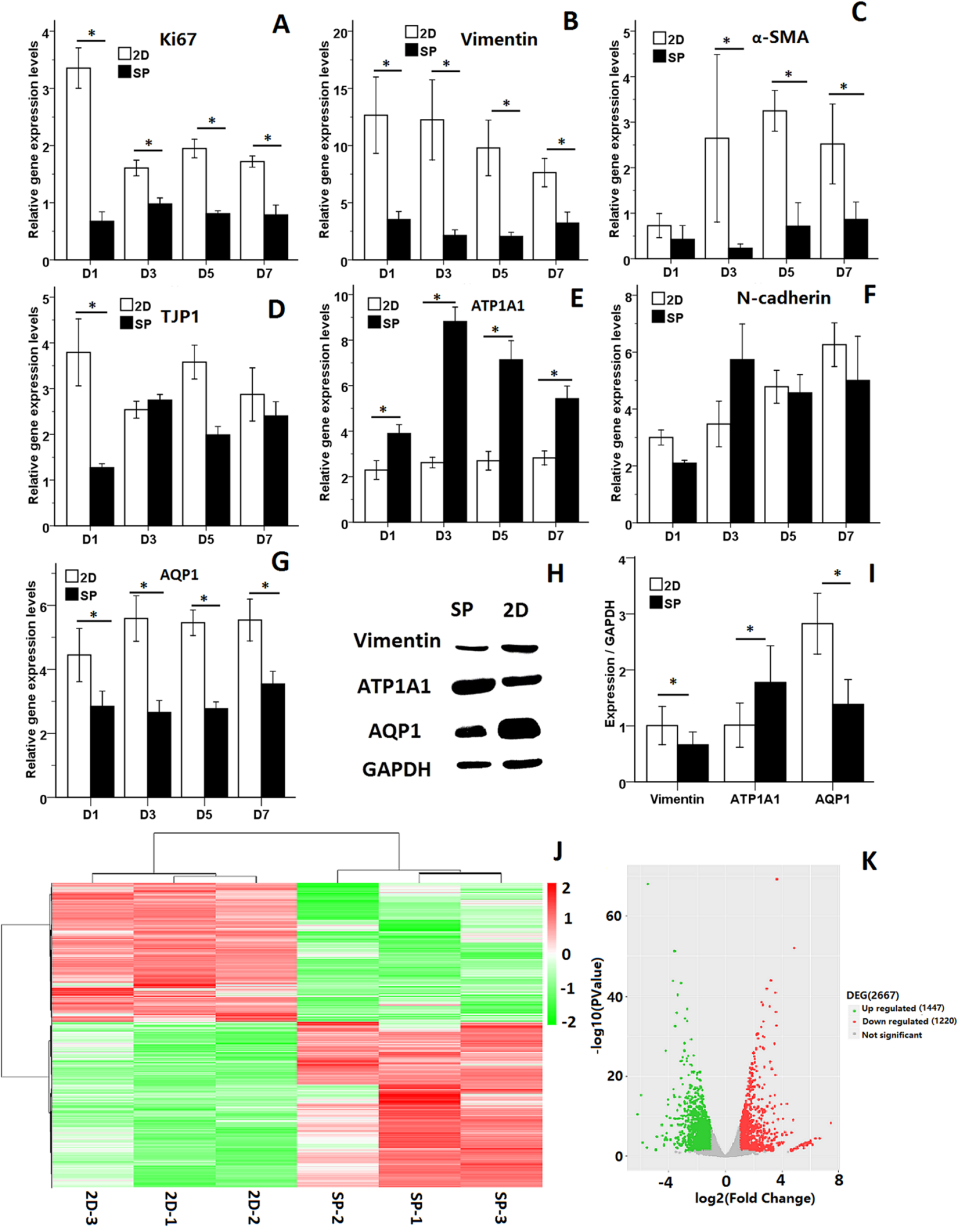
Impediment of EMT in CECs by biomimetic platforms of CEC spheroids. (**A**–**G**) QPCR analysis of proliferation marker and functional marker expression normalized to GAPDH. (**H**) WB of vimentin, ATP1A1, AQP1 and GAPDH in 2D and SP cultures. (**I**) Quantification of protein expression levels by Western blotting using Image J software. Differences with *P < 0.05 were considered statistically significant. (**J**) Hierarchical cluster analysis of gene expression based on log ratio RPKM data. (**K**) Differences in the gene expression profiles of 2D and SP cultures.

Total RNA was extracted from the CECs of traditional 2D monolayer cultures and from CEC cultures containing small spheroids (SP). The RT-PCR results showed that expression of a proliferation marker (*Ki67*), EMT-related markers (*Vimentin* and *α*-*SMA*) and CEC markers (*ATP1A1*, *AQP1*, *N*-*cadherin* and *TJP1*) was positive in both the 2D and the SP cultures (Fig. S5). However, the QPCR assay revealed a difference between the two groups. The QPCR results from CEC spheroids revealed significant down-regulation of gene expression for the proliferation marker *Ki67* on D1, D3, D5 and D7 (Fig. 4A). The EMT-related markers *Vimentin* and *α*-*SMA* were significantly down-regulated on D3, D5 and D7 (Fig. 4B,C), whereas there was significant up-regulation of the endothelial marker *ATP1A1* at those times (Fig. 4E). There was no consistent difference in the *TJP1* and *N*-*cadherin* expression levels in the two groups (Fig. 4D,F). The gene expression level of the CEC marker *AQP1* was significantly down-regulated (Fig. 4G). Western blot analysis also revealed significantly up-regulated expression of the ATP1A1 protein and significant down-regulation of the expression of Vimentin and AQP1 in cultured CEC spheroids on D3 (Fig. 4H,I).

We next investigated the global gene expression of 2D and SP cultures on D3 using RNA-seq. The Pearson correlation heatmap revealed that SP cultures showed a drastically different gene expression pattern than 2D cultures (Fig. S6). To identify differentially expressed genes (DEGs), these data were subjected to hierarchical clustering using the Pearson correlation as the distance metric (Fig. 4J). Compare to 2D libraries, 1447 genes were up-regulated and 1220 genes were down-regulated in the SP libraries by edgeR analysis (Fig. 4K). The GO enrichment analysis results showed that up-regulated DEGs were significantly enriched in biological processes (BP), including regulation of metabolic process, gene expression and biosynthetic processes (Fig. S7); the down-regulated DEGs were significantly enriched in cell division, migration, mitotic cell cycle, organelle fission and other functions (Fig. S8). This may explain the low level of expression of the cell proliferation marker Ki67 in SP. For molecular function (MF), the up-regulated DEGs were enriched in ion binding, protein kinase activity, cAMP response element binding and AMP-activated protein kinase activity (Fig. S7), and the down-regulated DEGs were enriched in ATP binding, protein binding, anion binding and other functions (Fig. S8), indicating that SP promotes the pump function of CECs. In addition, GO cell component (CC) analysis indicated that the up-regulated DEGs were significantly enriched in the cell part, organelles, and lumen (Fig. S7) and that down-regulated DEGs were enriched in genes related to cell division, the cytoskeleton, ECM, cell-substrate junctions, and other functions (Fig. S8). To identify the biological pathways that are active, we mapped the detected genes to reference pathways in the Kyoto Encyclopedia of Genes and Genomes (KEGG) (Fig. S9).

**Figure 5:**
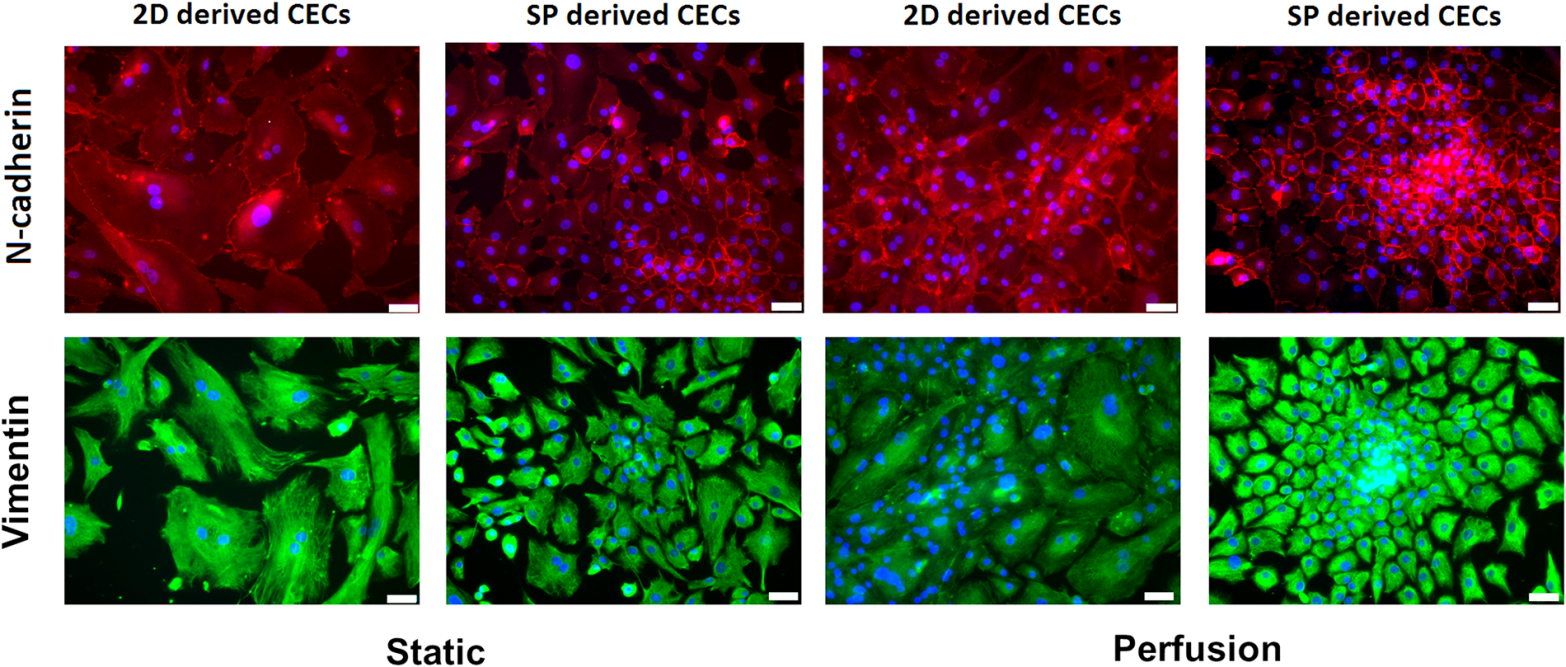
Phenotypic expression of CECs in the static and perfusion systems. Immunofluorescence staining for N-cadherin and vimentin in 2D- and SP-derived CECs in the static and perfusion systems. Cell nuclei were counterstained with DAPI. Scale bar: 50 µm.

Based on the results described above, small spheroids cultured for 3 days were directly reseeded on the glass in the perfusion system. Many of the cells in the spheroids migrated onto the culture glass from the adherent CEC spheroids. The spheroids disappeared, and the monolayer CECs completely covered the glass after D7. The representative immunofluorescence images shown in Fig. 5 demonstrate that CECs in both the static and perfusion systems were all positive for N-cadherin and vimentin on D5. However, the CECs in the static monolayer culture displayed an irregular, enlarged pattern, whereas CECs in the perfusion spheroid culture were arranged in confluent monolayers and were polygonal in shape with more compact and contact-inhibited features. It is interesting to note that the expression of N-cadherin in the static monolayer culture appeared as cytoplasmic diffuse staining around the nucleus, whereas N-cadherin in the perfusion spheroid culture was obviously located in the cytoplasmic membrane. The results demonstrated that perfusion spheroid culture promotes CEC cell-cell contact of the adherens junction (AJ)-related protein N-cadherin at the lateral cell borders. Taken together, the results of our characterization of spheroid-derived and 2D culture-derived CECs show that spheroid culture increases specific corneal endothelial expression, resulting in better maintenance of untransformed corneal endothelium in CEC spheroids in static culture. The use of biomimetic platforms that combine the use of the perfusion system and 3D spheroid culture impedes EMT in CECs, enhances CEC cell-cell contact, and promotes the generation of a healthy corneal endothelial layer.

### The perfusion system and 3D spheroid culture promote the growth of tissue-engineered CECs on decellularized corneal scaffolds

H&E staining showed that cells were eliminated in our decellularized bovine cornea (Fig. S10B,C), whereas normal bovine corneas contain corneal epithelial, stromal and endothelial cells (Fig. S10A). SEM evaluation showed the smooth surfaces of Bowman’s layer (Fig. S10D) and Descemet’s membrane layer (Fig. S10F) as well as the rough surfaces of the acellular stromal lamellae (Fig. S10E). Those results show that the cells of the cornea were removed using our short-term chemical-frozen decellularization.

**Figure 6:**
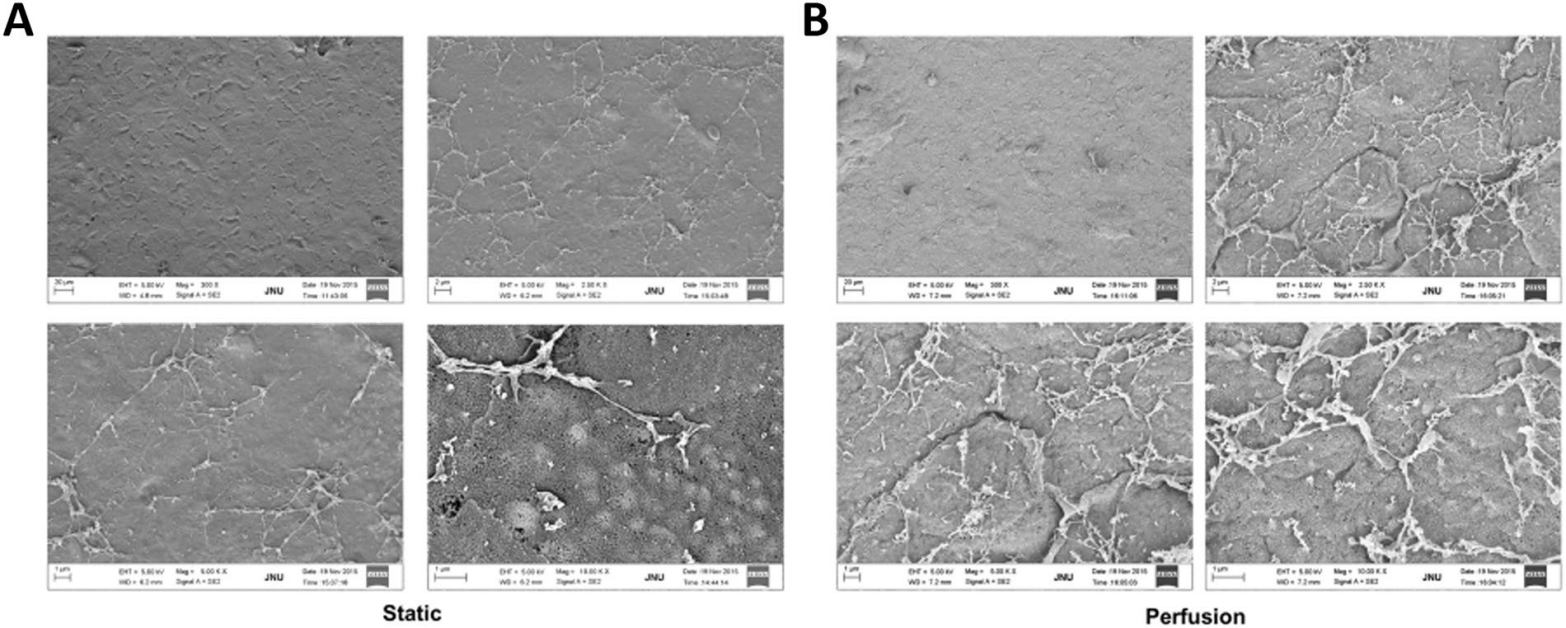
CEC spheroids cultured on decellularized corneal scaffolds in the static and perfusion system. SEM images of CECs on decellularized corneal scaffolds in the static (**A**) and perfusion (**B**) systems.

Based on our characterization of CECs obtained using the perfusion system and 3D spheroid culture, we constructed tissue-engineered corneal endothelial layers on decellularized corneas under the same conditions. Small spheroids cultured for 3 days were reseeded on the Descemet’s membrane surface of decellularized corneas. After incubation for 1 week under static conditions to allow the spheroids to adhere firmly to the decellularized corneas, they were transferred to perfusion culture for 1 week. SEM images revealed that the spheroids disappeared and that a monolayer of spheroid-derived CECs completely covered the surface of the decellularized corneas. Interestingly, spheroid-derived CECs maintained in the static condition were arranged in flat monolayers with rare extracellular matrix (ECM) and microvilli distributed over the smooth surface of the cell layer (Fig. 6A), while the monolayer derived from spheroids in the perfusion system was characterized by rough surface topography with many ECM fibres and more microvilli (Fig. 6B). Thus, the perfusion spheroid culture in conjunction with decellularized corneal scaffolds promoted the generation of CEC monolayers and neo-synthesized ECM formation.

### The perfusion system and 3D spheroid culture promote tissue engineering of CECs on collagen sheets

**Figure 7:**
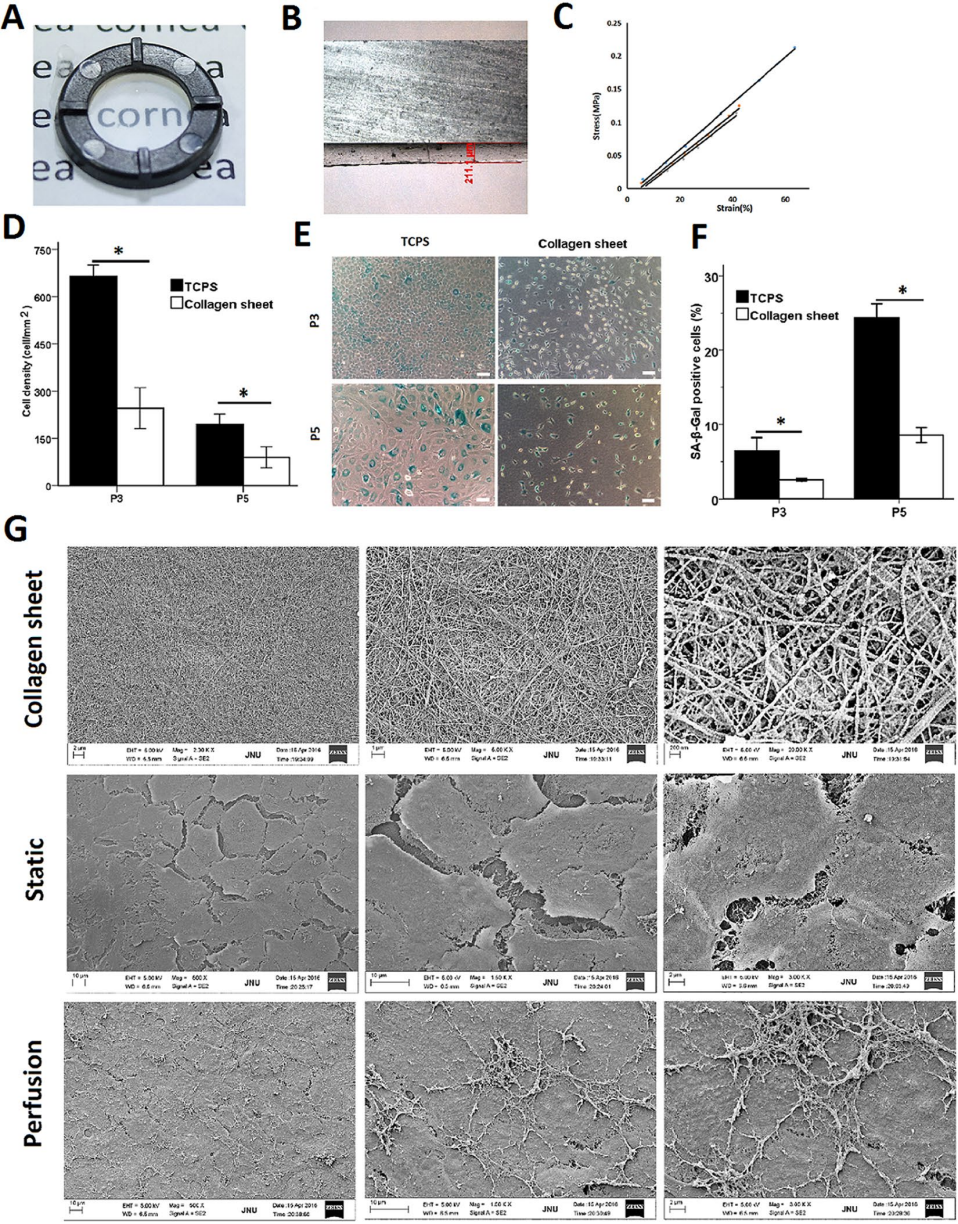
Characterization of the flat collagen sheet and the perfusion system for corneal tissue engineering. (**A**) Photograph of a flat collagen sheet. (**B**) Representative section of the collagen sheet. (**C**) Tensile stress-strain curve for each sample. (**D**) Cell density was calculated based on the total number of cells in each square millimetre. (**E**) Cells at different passages on TCPS and on collagen sheets were stained to detect SA-β-Gal activity. Scale bar: 100 μm. (**F**) The senescence level of the CECs was evaluated based on the ratio of SA-β-Gal-positive cells to total cells. (**G**) SEM images of the collagen sheet prepared under vitrification conditions and representative images of CECs on collagen sheets in static and perfusion culture. Differences with *P < 0.05 were considered statistically significant.

The ability to expand CECs on collagen sheets provides an opportunity to generate suitable cells for therapeutic applications. As a first step in evaluating this potential, we tested the properties of collagen sheets. Collagen gels were rehydrated and converted into flat transparent collagen sheets 200 ± 50 µm in thickness (Fig. 7A,B). The stress-strain assay showed that the curves for each sample (n = 3) were clustered and that all formulations were linearly elastic up to 40% strain (Fig. 7C). To evaluate potential changes in the senescence of CECs grown on collagen sheets, we conducted a senescence assay of the passaged cells. First, we found that CECs expanded on TCPS formed monolayers of significantly higher density at passage 3 (P3) (664 ± 35 cells/mm^2^) and passage 5 (P5) (196 ± 32 cells/mm^2^) on D6 than CECs expanded on collagen sheets at P3 (246 ± 64 cells/mm^2^) and P5 (89 ± 33 cells/mm^2^) (Fig. 7D). However, as assayed by SA-β-gal staining, senescence was significantly decreased in CECs at P3 and at P5 on collagen sheets on D6 compared with those on TCPS (Fig. 7E). The percentages of SA-β-Gal-positive cells at P3 on TCPS and on collagen sheets were (6.50 ± 1.73)% and (2.58 ± 0.16)%, respectively. Moreover, serial passage dramatically increased the percentage of SA-β-Gal-positive cells at P5 on TCPS (24.39 ± 1.82)% and on collagen sheets (8.57 ± 1.02)% (Fig. 7F).

We used small spheroids cultured for 3 days and reseeded them on collagen sheets. After incubation for 1 week in the static condition, they were maintained under perfusion culture for another week. CEC spheroids disappeared, and the monolayer of spheroid-derived CECs on the surface of the collagen sheet in the perfusion culture reached 100% confluence, while their counterparts on the collagen sheet in static culture reached approximately 80–85% confluence. Immunofluorescent staining showed that vimentin, N-cadherin, AQP1 and F-actin were expressed in spheroid-derived CECs on collagen sheets both in the static system and in the perfusion system. N-cadherin and F-actin were arranged in polygonal and hexagonal patterns indicative of lateral cell border staining (Fig. S11). SEM images showed a relatively smooth surface on the collagen sheet. However, images from higher magnifications revealed that the collagen sheet displayed a rough surface with a series of collagen fibres arrayed irregularly (Fig. 7G, top panel). In addition, SEM images obtained at low magnification showed that spheroid-derived CECs in static culture manifested as a crack-like monolayer, whereas images from higher magnifications showed that spheroid-derived CECs covered the collagen sheet surface with loose cell-cell junctions (Fig. 7G, middle panel). In the perfusion system, however, spheroid-derived CECs completely covered the collagen sheet and displayed tight intercellular junctions and numerous microvilli. Higher magnification showed that the apical surfaces of spheroid-derived CECs on the collagen sheet were covered by an extensive newly synthesized ECM (Fig. 7G, bottom panel). These results demonstrate that our thin collagen sheet is transparent and has a certain mechanical strength. CECs expanded on the collagen sheet show lower percentage of senescent cells than CECs expanded on TCPS. At the same time, the collagen sheet enhances the monolayer growth and promotes ECM formation by spheroid-derived CECs maintained in the perfusion system with continuous supplemental nutrition.

### Tissue-engineered CECs grown on curved collagen sheets in the perfusion system under controlled pressure

**Figure 8:**
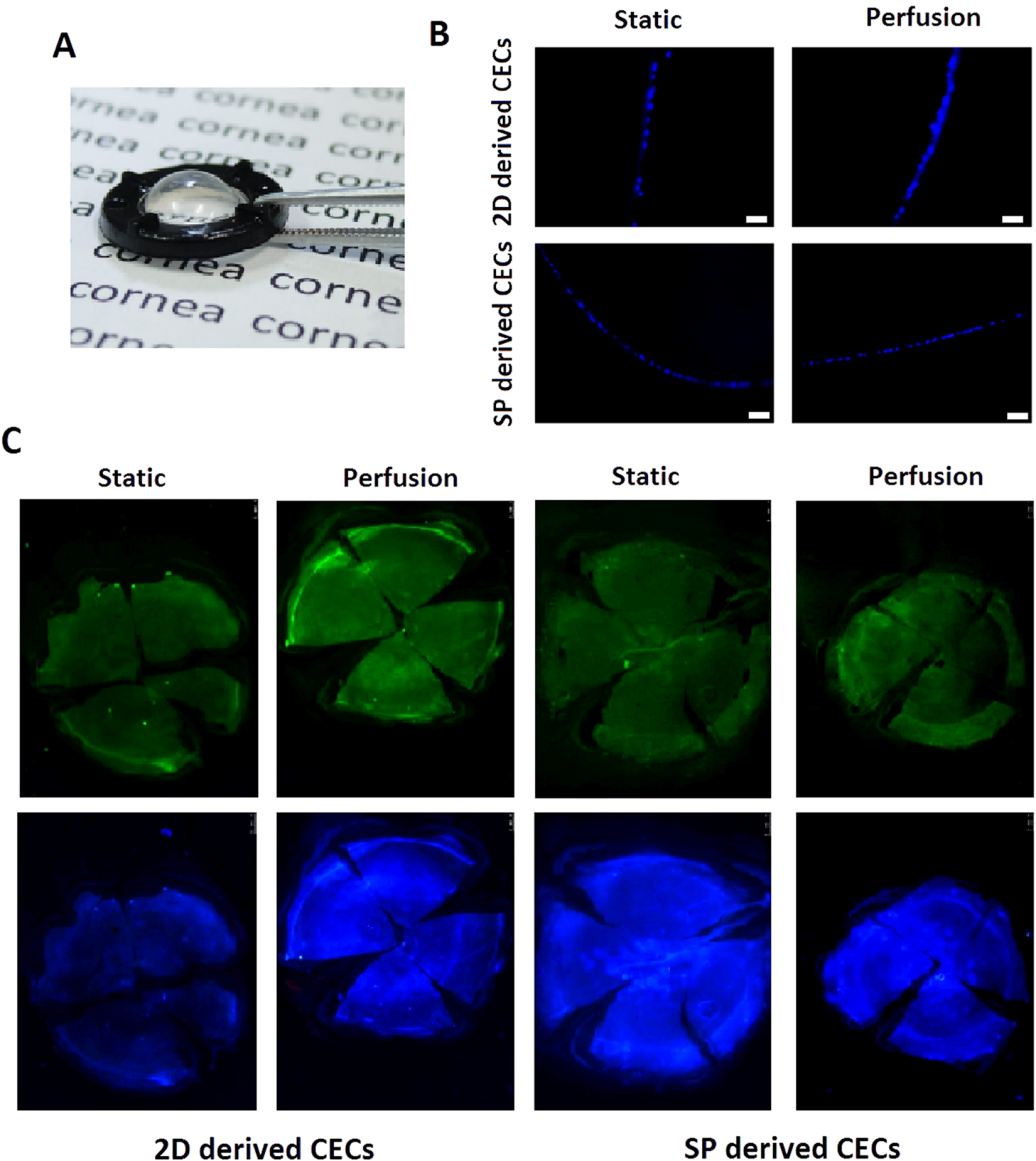
Tissue-engineered CECs on curved collagen sheets in the perfusion system under controlled pressure. (**A**) Photograph of a curved collagen sheet. (**B**) Immunohistochemical analysis of cryosectioned spherically curved collagen sheet by DAPI. Scale bar: 50 µm. (**C**) Representative fluorescence microscopy images of CEC monolayers stained for F-actin and with DAPI on spherically curved collagen sheets in the static and perfusion systems.

To obtain a CEC biomimetic environment to generate engineered tissue, we used a curved scaffold and a perfusion system with controlled pressure. To construct the biomimetic scaffold, we developed a collagen sheet with spherical curvature, produced with a spherically curved mould 8 mm in diameter. The collagen sheet was transparent and displayed spherical curvature (Fig. 8A). In addition, we were able to control and measure the pressure in the perfusion system, as shown in the schematic illustration in Fig. S1A. To obtain an environment that mimics physiological intraocular pressure, we maintained the pressure at 15 mmHg during the perfusion culture period (Fig. S1B). DAPI staining of cryosectioned curved collagen sheets with 2D- or SP-derived CECs in static or perfusion culture at 15 mmHg pressure showed the presence of a curved CEC monolayer after 1 week of culture (Fig. 8B). Whole-mount DAPI and anti-F-actin staining (blue fluorescence and green fluorescence, respectively) also demonstrated that these CECs formed a monolayer at the 1-week time point (Fig. 8C). Our findings show that a spherically curved collagen sheet and perfusion system with controlled pressure may be used to construct biomimetic tissue-engineered corneal endothelial layers *in vitro*.

## Discussion

The expansion of CECs for tissue engineering is usually performed under 2D static conditions. However, although conventional cultured CECs have been observed to undergo one or two population doublings *in vitro*, they rapidly become senescent or undergo EMT to a fibroblastic phenotype^36^. Along with the understanding that the entire context of a cell’s microenvironment is important in tissue engineering, it is generally realized that it is necessary to reconstruct dynamic *in vivo* environments in *in vitro* experimental systems. Recently, the development and use of biomimetic platforms in which the presence and levels of regulatory molecules (oxygen and nutrients), other cells (3D context and cell-cell contacts), ECM (topology and stiffness), and physical factors (flow shear and compression) are controlled are increasingly being applied to the generation of engineered tissues^38^. The use of dynamic bioreactors could influence major cellular events such as differentiation, proliferation, viability and the cell cycle. To improve the environment in which cells and developing tissues can be maintained under *in vitro* conditions, the MINUSHEET perfusion culture system was developed. In this perfusion system, a constant flow of culture medium provides seeding cells and tissues with a constant supply of fresh nutrients and respiratory gases, making it possible to produce a variety of specialized tissues of the high cell biological quality that is urgently needed in tissue engineering^18^. The use of a perfusion system benefits cells by providing a continuous supply of nutrients and constant removal of metabolic waste. Therefore, it is suitable for the generation and long-term maintenance of various types of specialized tissues. Wu and co-investigators reported that perfusion culture can be used to reconstruct an auto-lamellar cornea with favourable morphological characteristics and satisfactory physiological function^39^. Wang *et al*. reported that the density of bone marrow mesenchymal stem cells (MSCs) in the perfusion system used in their study remained at a high level over the period of induction of hepatocytic differentiation and was almost two-fold the density of cells in the static system at the end of the induction period. For cells in scaffolds, perfusion induction was more effective than static induction^40^. Many published papers report that dynamic perfusion culture contributes to better growth of various cells, including endothelial progenitor cells^41^, human endometrial stromal cells^42^ and dermal fibroblasts^43^, than static culture. Moreover, perfusion bioreactors have been introduced and shown to enhance cellular access to oxygen and nutrients. The use of perfusion-based bioreactor culture can significantly improve the access of cells to oxygen, enhancing the viability and contractility of the engineered tissues^44^.

The results of our study are consistent with previous reports. First, we showed that the dissolved oxygen concentration in perfusion culture, as measured by an optical fibre oxygen fluorescence microsensor, is higher than that in static culture. The use of such microsensors offers a new methodology for the measurement of dissolved oxygen based on fluorescence quenching^45–47^. Fluorescence microsensors offer some advantages over the traditional Clark electrode^48^, such as their small size and their suitability for remote sensing and multiplex microsensor networking. Optical fibre oxygen fluorescence microsensors do not consume oxygen and can be used to measure oxygen in both the gas and liquid phases. Their potential rapid response time makes them suitable for continuous dynamic pO2 monitoring^49^. Second, by comparing the survival and proliferation of CECs in perfusion and static cultures, we showed that the perfusion condition increased the percentage of EdU-positive nuclei and cells entering the S phase and resulted in a higher efficiency of CEC expansion and further development of the tissue-engineered CECs. Therefore, the microenvironment provided by the perfusion system was more favourable than that provided by the static system for CEC expansion *in vitro*. Third, we found that the perfusion system helps maintain the AQP1 and ATP1A1 phenotypic marker characteristics of CECs. The perfusion environment can support the expression of the CEC phenotypic markers AQP1, vimentin and N-cadherin, even in CECs passaged on 2D culture TCPS after perfusion culture. Aquaporin 1 (AQP1) is the major water channel isoform in CECs and is essential for CEC proliferation and migration via the ERK signalling pathway^50^. The ATP1A1 pump is an integral membrane protein expressed in the basolateral membrane of CECs that is responsible for continuously pumping Na^+^ out of the cell and K^+^ into the cell, utilizing energy from ATP. As such, it plays important roles in preserving corneal dehydration and transparency^51^. Vimentin is a major component of the mesenchymal cell cytoskeleton and is also expressed in human CECs^52^. Due to its neuroectodermal origin, the corneal endothelium represents a special case with respect to the expression of cadherin. N-cadherin, but not E-cadherin, plays a central role as an adherent junction protein in human CECs^53^. Interestingly, from SEM microscopic studies, we found that CECs in perfusion culture exhibited rough topography with many microvilli, unlike the smooth surface profiles exhibited by cells maintained in static culture. Based on our results, we propose that the use of a perfusion system that provides continual supplementary nutrition promotes CEC proliferation and phenotypic maintenance. Additionally, augmenting the concentration of dissolved oxygen in the perfusion system may be involved in such an effect.

Spheroid size influences cell viability and phenotypic outcome^54^. In the present study, QPCR revealed significant up-regulation of the endothelial markers ATP1A1, AQP1 and N-cadherin, the proliferation marker Ki67 and the mesenchymal markers vimentin and α-SMA in small spheroids compared to spheroids with larger diameters. It is possible that the cells in small spheroids are able to obtain more nutrients and oxygen and that as a result they show better growth status. Huang *et al*. found that dermal papilla (DP) spheroids of larger size displayed slightly decreased viability on D5^55^. A large spheroid core is often deficient in oxygen and/or nutrients and shows excessive accumulation of catabolites and low pH^56, 57^. We found that CECs in spheroid culture displayed enhanced expression of the corneal endothelial marker ATP1A1. In addition, the enhanced gene expression of ATP1A1 in CEC spheroids was coincident with the results of GO enrichment of target genes for molecular function obtained by RNA-Seq analysis. The results of the latter assay indicated that protein kinase activity, cAMP response element binding and AMP-activated protein kinase activity were significantly up-regulated. Wigham *et al*. demonstrated that increased concentrations of cAMP activate the ATP1A1 activity and enhance the pump activity of CECs^58^.

QPCR assays of CEC spheroids revealed significant down-regulation of gene expression for the proliferation marker Ki67. RNA-Seq analysis also confirmed the enhanced proliferation of CEC spheroids. GO enrichment of target genes in CEC spheroids for biological process demonstrated that down-regulated DEGs were significantly enriched in cell division, mitotic cell cycle, organelle fission and other functions. In the cellular component, down-regulated DEGs were significantly enriched in cell-division-related genes (spindle, kinetochore, condensed chromosome and others). The pathway enrichment analysis also demonstrated that the cell cycle was significantly down-regulated in CECs in spheroids compared with CECs in 2D culture. These results suggest that CECs in spheroids have a low rate of proliferation and cell division, a finding that is consistent with other reports. For example, Cheng *et al*. found that adipose-derived stem cells (ASCs) almost cease to proliferate upon spheroid formation, although later expansion of dissociated spheroid cells in monolayer cultures exhibited higher proliferative activity^59^.

EMT in CECs is the process by which endothelial cells lose their specific markers and acquire mesenchymal characteristics. EMT usually leads to fusiform morphology, transformed phenotype, and abnormal function of cultured CECs. Thus, it is clear that cultured CECs that have undergone EMT will not be of use in regenerative medicine. For the use of cultured CECs in regenerative medicine to be practical, EMT must be antagonized during the culture process^53^. Recently, spheroid culture has gained increased attention for its ability to improve cellular pluripotency^60^, functional maintenance^61^ and 3D bio-printing^62, 63^. Spheroid culture enables cells to assemble and interact under native conditions and to form biomimetic 3D environments that enhance high-density growth, cell-cell contact and cell-matrix interactions^64^. Therefore, we used spheroid culture as a biomimetic means to improve CEC growth and to further construct tissue-engineered corneal endothelial layers. We found that CEC spheroid culture can reverse the gene and protein expression levels of the EMT-related markers vimentin and α-SMA. The results of RNA-Seq analysis of differentially expressed genes (DEGs) of CECs in 2D and SP culture are consistent with these characteristics. GO enrichment of target genes from CEC spheroids demonstrated that down-regulated DEGs in the cellular component were significantly enriched in the cytoskeleton, ECM, cell-substrate junction, and other functions. We propose that spheroid culture of CECs decreases the expression of genes related to the actin cytoskeleton, ECM components and CEC-ECM adhesion, components that are closely associated with the regulation of cellular activities such as EMT. Previous studies reported that EMT is involved in the formation of cellular protrusions and the reorganization of the actin cytoskeleton. Changes in the ECM are able to induce EMT. Increased ECM density can promote EMT by enhancing cell–matrix adhesions and weakening cell–cell adhesion^65, 66^. In our study, the pathway enrichment scores obtained by RNA-Seq for 2D and SP CEC cultures showed changes in the signalling pathways involving thyroid hormone and Hippo. Lin *et al*. showed that thyroid hormone (T3)/thyroid hormone receptor (TR) signalling up-regulated the expression of cell EMT-related proteins, MMP9, p-mTOR, p-STAT3, p-AKT and p-ERK1/2^67^. The downstream transcriptional co-activators of the Hippo pathway, YAP and TAZ, have been implicated in both physiological EMT and pathological EMT^68^. From the above data and reports, we deduce that the underlying mechanism through which EMT is impeded in CEC spheroids involves down-regulation of the thyroid hormone and Hippo signalling pathways.

CECs cultured as spheroids in the perfusion system appeared as confluent monolayers with a polygonal shape, a more compact appearance and more AJ-related N-cadherin protein compared with the lateral cell border pattern. Our data revealed, for the first time, that *in vitro* biomimetic platforms using a perfusion system and 3D spheroid culture hinder EMT in the corneal endothelium. Moreover, CEC spheroids displayed significant down-regulation of gene expression for the proliferation marker Ki67, indicating a lower proliferation of the CEC cells in spheroids than in 2D culture. However, the perfusion system was able to promote CEC proliferation, indicating that perfusion culture can overcome the minor drawback of somewhat slow growth associated with spheroid culture. Therefore, use of the perfusion system in conjunction with spheroid culture improves CEC monolayer formation.

To explore whether spheroid culture in conjunction with the perfusion system has potential utility in tissue engineering, we constructed CEC layers. The use of substrates not only provided mechanical support during the transplantation of *ex vivo*-engineered CEC sheets but also created a favourable microenvironment for cellular activity^6^. Perfusion bioreactors have been shown to enhance the access of cells to oxygen and nutrients as well as the homogeneity of neo-synthesized ECM in 3D scaffolds^69, 70^. The results of our study are consistent with these findings. We used a natural decellularized corneal matrix and collagen sheets as scaffolds for CEC tissue engineering. We found that the combination of perfusion spheroid culture and decellularized corneal scaffolds or collagen sheets promotes the formation of neo-synthesized ECM, microvilli and CEC monolayers. We also tested the senescence of the CECs on the scaffolds. First, we found that it is difficult to observe SA-β-gal staining in CECs on decellularized corneal scaffolds because hydration of the decellularized corneal matrix during culture interferes with the clear microscopic imaging of stained cells. In addition, there are some difficulties associated with SA-β-gal staining and evaluation of CEC spheroids at P3 and P5. Therefore, we only compared the senescence of dissociated CECs on collagen sheets with that of cells cultured on TCPS. At P3 and P5, CECs on collagen sheets exhibited less staining for the senescence marker SA-β-gal than CECs on TCPS. Collagen is the major protein constituent of the ECM and is a commonly used component in tissue scaffold construction^71^. Our transparent collagen sheet can also be made into a spherically curved shape to fit the corneal curvature. Zhang *et al*. created a cornea-shaped scaffold using collagen^72^. Yoshida *et al*. developed a transparent porcine atelocollagen with a curved shape and adequate mechanical strength. They showed that the curved shape is important for adhesion of the CEC carrier to the posterior surface of the cornea^33^. Kimoto *et al*. also demonstrated that a spherically curved gelatin hydrogel sheet with CECs achieves close adhesion to the posterior corneal surface^73^. In the present study, we showed that curved tissue engineering of the corneal endothelial layer can be accomplished in the perfusion system at 15 mmHg pressure. The normal range of intraocular pressure is 10–21 mmHg. Therefore, we can construct biomimetic tissue-engineered corneal endothelial layers from curved collagen sheets in a perfusion system that is maintained at physiological intraocular pressure.

In this study, we established biomimetic platforms to promote the construction of tissue-engineered corneal endothelial layers *in vitro*. First, we used a perfusion system that provides a higher dissolved oxygen concentration in the medium and enhances CEC proliferation compared to static culture. Second, we used a spheroid culture, which resulted in a lower EMT change. Third, we showed that use of the perfusion system in conjunction with a spheroid culture promotes monolayer formation by untransformed CECs with normal cell-cell contact junctions. Fourth, perfusion CEC spheroid cultures on decellularized corneal scaffolds and collagen sheets were shown to promote the generation of CEC monolayers and neo-synthesized ECM formation. Fifth, we showed that perfusion CEC spheroid cultures maintained on curved collagen sheets under a controlled physiological intraocular pressure can generate CEC monolayers. In summary, our comprehensive biomimetic platforms involving a dynamic perfusion system with or without a controlled pressure, spheroid culture, decellularized corneal scaffolds, and flat or curved collagen sheets provide a suitable microenvironment for the maintenance of the normal CEC physiological context of CECs, thereby improving corneal endothelial tissue engineering and regeneration.

## Methods

### Ethics statement

Bovine eyes were acquired from a local abattoir (Shipai, Guangzhou, China). The study was conducted according to the Association for Research in Vision and Ophthalmology Statement on Using Animals in Ophthalmic and Vision Research and the guidelines of the Animal Experimental Committee of Jinan University, Guangzhou, China.

### Isolation and culture of bovine CECs

Bovine CECs were cultured as previously described^74^. Briefly, the corneal tissues were washed three times with phosphate-buffered saline (PBS) containing 2% penicillin-streptomycin and 50 μg/ml gentamicin. The Descemet’s membrane was peeled away from the posterior surface of the tissue with a sterile surgical forceps under a dissecting microscope. The strips were incubated in trypsin/EDTA at 37 °C for 8–10 min. The cells were centrifuged (300× g, 5 min), seeded into a 6-well tissue-culture-treated polystyrene (TCPS) plate and cultured in low-glucose DMEM supplemented with 10% FBS and 1% penicillin-streptomycin in a 37 °C incubator under 5% CO_2_.

### Culture of CECs in the perfusion system

Bovine CECs were seeded at a density of 0.7 × 10^4^ cells/well on glass slides mounted in tissue carriers and allowed to remain for 1 day. The CECs on the glass slides in the tissue carriers were then directly transferred into the MINUSHEET flow perfusion culture container for an additional 5 days of culture. The MINUSHEET perfusion system is illustrated in Fig. 1A,B. Continuous perfusion with the culture medium was accomplished using a slowly rotating peristaltic pump (ISMATEC, IPC N8, Wertheim, Germany) that was able to deliver pump rates of 1 ml per hour. During perfusion culture, CECs on the glass slides were always exposed to fresh medium from a storage bottle; the waste medium was collected in a separate waste bottle and was not re-circulated. The morphology of cells transferred out of the perfusion culture container at various times was observed using phase-contrast microscopy. To compare the proliferation and viability of cells cultured in the perfusion system with those of cells cultured under static conditions, the cells were dissociated with 0.25% trypsin/EDTA and counted using a hemocytometer at various intervals during the 5-day period.

### Oxygen measurement

To measure the change in the dissolved oxygen concentration in the culture medium of the perfusion system, we used an optical fibre oxygen fluorescence microsensor at an atmospheric pressure of 101.3 kPa and a temperature of 297.13 K. Figure 1C shows a schematic of the sensor system. Figure 1D shows a photographic image of the microsensor. In this system, the excitation provided by an LED with a central wavelength of 405 nm is fed to the microsensor, and the emitted luminescence from the microsensor is transmitted to the spectrometer through the fibre coupler. The spectrum of the luminescence is detected by the spectrometer (Ocean Optic; USB2000).

The fluorescence intensity is directly related to the oxygen concentration according to the Stern–Volmer equation (1)^75^:

1 $$\frac{{I}_{0}}{I}=1+K\cdot [{O}_{2}]$$

where *I*_0_ and *I* represent the steady-state luminescence intensities in the absence and presence of O_2_, respectively, *K* is the Stern–Volmer quenching constant, and [O_2_] is the O_2_ concentration. In liquid, the O_2_ concentration is the dissolved oxygen concentration.

On D3 of CEC culture, the fibre optic microsensor probe was positioned in the medium of the static and dynamic cell cultures successively for 75 s. For comparison, the dissolved oxygen in the unused medium and in the waste medium of the perfusion were measured at the same time. The relative fluorescence intensity was used to analyse the dissolved oxygen concentration. Prior to each measurement, the fibre optic probe was washed with H_2_O to avoid cross-contamination. To test the stability of the microsensor, the relative fluorescence intensity with the microsensor positioned in H_2_O was also measured.

### EdU labelling assay

Bovine CECs were seeded on glass slides at 0.7 × 10^4^ cells/well and were allowed to attach for 1 day. Next, CECs were continually cultured in the perfusion or static system for another 3 days. The EdU labelling assay was conducted according to the manual of the EdU labelling/detection kit (Ribobio, Guangzhou, China). Samples were then observed and photographed under a fluorescence microscope. The percentage of EdU-positive cells was calculated, respectively, from five random fields in three wells.

### Flow cytometry

Flow cytometry was used to determine the cell cycle distribution in bovine CECs. Briefly, CECs were seeded on glass slides at 5 × 10^4^ cells/well and allowed to attach for 1 day. The CECs were then continually cultured in the perfusion system or the static system for another 3 days. The cell cycle distribution was then analysed by PI flow cytometry (FACS Calibur, BD, USA) as previously described^74^.

### Immunofluorescence

After fixation in 4% (wt/vol) paraformaldehyde for 15 min, the CECs were washed three times with PBS, permeabilized with 0.1% Triton X-100 in PBS for 10 min and incubated with PBS containing 5% BSA for 30 min. The cells were then incubated overnight at 4 °C with primary antibodies, including rabbit polyclonal anti-ATP1A1 antibody (1:200; Santa Cruz, USA), rabbit polyclonal anti-AQP1 antibody (1:200; Santa Cruz, USA), anti-N-cadherin (1:200; BD, USA), and anti-vimentin (1:200; Proteintech, USA), followed by washing three times in PBS and incubation with secondary antibody for 1 h at room temperature. For F-actin staining, FITC/phalloidin (Yeasen, China) was used according to the manufacturer’s instructions. For collagen sheet staining, single CECs or CEC spheroids on flat or spherically curved collagen sheets were stained by DAPI for 10 min. The collagen sheet was then cut into four quadrants. Imaging was performed using a fluorescence stereo microscope (M165 FC) and an EL6000 external light source (Leica Microsystems).

### SA-β-Gal staining and cell viability assays

The detection of senescence-associated β-galactosidase (SA-β-Gal) activity was performed using a commercial senescence staining kit (Beyotime Biotechnology, China) according to the manufacturer’s instructions. Briefly, single CECs were seeded into 12-well TCPS prepared with and without coating with collagen sheets and cultured for 6 days. The cells were then fixed in SA-β-Gal fixing solution for 15 min, stained with working solution overnight at 37 °C, and imaged using phase-contrast microscopy. The cell viability assay was performed with a viability/cytotoxicity assay kit (Life Technologies, USA) for live/dead cells according to the manufacturer’s instructions.

### Reverse transcription-polymerase chain reaction (RT-PCR) analysis and quantitative polymerase chain reaction (qPCR)

Total RNA from CECs derived from spheroids and monolayer cultures was extracted using TRIZOL reagent. The cDNA was synthesized using a reverse-transcriptase reagent kit (TOYOBO, Japan) according to the manufacturer’s instructions. Gene-specific primers were synthesized by Sangon Biotech (China); the sequences of the primers are listed in Table S1. For qPCR experiments, gene expression was analysed by real-time PCR (Bio-Rad CFX96TM, USA) with two or three replicates per sample. The GAPDH gene was used as an internal control. Expression changes in the gene transcripts for each sample were calculated using the 2^−∆∆Ct^ method. The results from three independent experiments were statistically analysed.

### Scanning electron microscopy (SEM)

SEM was used to observe the surface ultrastructure of acellular corneal scaffolds and collagen sheets with and without CEC spheroid seeding. Briefly, the samples were fixed in 2.5% glutaraldehyde for 2 h and washed 3 times for 15 min each time in PBS. After dehydration in increasing concentrations of ethanol (70, 80, 90, 100, 100, and 100%) for 10 min each time, the specimens were transferred to isoamyl acetate for 30 min, subjected to critical point drying, coated with a gold-palladium alloy and viewed by SEM on a JSM-T300-SEM instrument (JEOL Technics Co. Ltd., Tokyo, Japan).

### Generation of cell spheroids in 3D Petri dishes

CEC spheroids were established in agarose 3D Petri dishes as described in our previous study^29^. Briefly, cell suspensions containing precisely measured numbers of cells were carefully seeded into the microwells of agarose dishes and were allowed to stand for 10 min to promote cell deposition. The cells were then incubated at 37 °C in a 5% CO_2_ incubator. The medium was changed every two days. CEC spheroids were formed, followed by cell aggregation and self-assembly; the spheroids were subsequently collected by flushing them out of the microwells gently with a pipette for further culture and experiments.

### RNA-Seq and analysis

Total RNA was extracted from 2D and SP cultures on D3 using TRIZOL reagent. After library construction and sequencing, we calculated reads as the number of reads per kilobase of exon model per million mapped reads (FPKM) to obtain normalized gene expression levels. We mapped the original RNA-seq to the reference transcriptome sequence using FANSe2 as previously described^76^. The correlation coefficients between gene expression levels were calculated and plotted as a correlation heatmap. Gene ontology (GO) analysis was performed using TopGO software (version 2.18.0), and comparisons between the two groups were made used Fisher’s exact test. Pathway enrichment analysis was primarily based on the Kyoto Encyclopedia of Genes and Genomes (KEGG) database. KOBAS software (kobas2.0-20150126) was used, and comparisons between the two groups were made using the hypergeometric test.

### Preparation of decellularized corneal scaffolds

Decellularized corneal scaffolds were established as previously described with minor modifications^29^. Briefly, bovine corneal samples were excised from fresh eyeballs and rinsed 3–5 times in PBS containing gentamicin sulphate. The corneal epithelium, including the posterior corneal stroma and the anterior corneal stroma, was stripped away with sterile surgical forceps and cut into lamellae (approximately 0.35 mm thick and 8.0 mm in diameter) using a biopsy punch under sterile conditions. The lamellae were immersed in a solution containing 0.5% Triton X-100 and 20 mM NH_4_OH for 30 min. After three additional rinses with PBS, the lamellae were preserved at −80 °C for 3 days and then transferred to 100% glycerol and stored at 4 °C. Prior to use, the decellularized corneal scaffolds were rehydrated in PBS.

### Immunoblotting

Total protein was extracted using RIPA lysis buffer containing the protease inhibitor PMSF (Beyotime, China). The proteins in the extract were separated by SDS-PAGE and transferred to PVDF membranes (Millipore, USA). Next, the membranes were incubated overnight at 4 °C with the following primary antibodies: rabbit polyclonal anti-vimentin (1:2000; Proteintech, USA); rabbit polyclonal anti-AQP1 (1:3000; Santa Cruz Biotechnology, USA); rabbit polyclonal anti-ATP1A1 (1:500; Santa Cruz Biotechnology, USA); and rabbit polyclonal anti-GAPDH (1:3000; Proteintech, USA). After washing, the membranes were incubated with HRP-conjugated secondary antibodies at room temperature for 1 h, and immunostained bands were visualized using enhanced chemiluminescence detection reagents (Pierce, Rockford, IL, USA).

### Preparation of collagen sheets and the stress-strain assay

Collagen sheets were prepared as previously described with modifications^77^. Briefly, a ring-shaped sterilized nylon membrane with an inner and outer diameter of 23 and 33 mm, respectively, was inserted into a polystyrene culture dish with a diameter of 35 mm. Equal volumes of 0.5% type-I collagen solution and culture medium [DMEM/F12 containing 10% FBS and 1% penicillin-streptomycin] were mixed, and 3.0 ml of the mixture was poured into the culture dish. The culture dish was incubated at 37 °C to complete gelation of the collagen. The collagen gel was then aseptically dried and converted into a flat collagen sheet. To imitate the posterior corneal curvature, a spherically curved mould with a diameter of 8 mm was placed on the collagen gel; this resulted in curvature of the collagen gel, which was then further dried to form a curved collagen sheet. The nylon frame provided support for the collagen gel, making it possible to easily separate the collagen sheet with tweezers. A dynamic mechanical analyser (Q800, TA instrument, USA) was used to evaluate the stress-strain on the collagen sheet in the horizontal direction. The results obtained from three independent specimens were statistically analysed.

### Examination of frozen sections

The CEC sheets on the spherically curved collagen sheets were fixed in 4% (wt/vol) paraformaldehyde for 15 min and washed in PBS. The structures were incubated at 4 °C in OCT compound solution for at least 4 h and then embedded in OCT compound at −80 °C. Frozen sections were cut at a thickness of 10 μm, placed on microscope slides and stained with DAPI. The sections were then stained and observed under a fluorescence microscope.

### Determination of the real-time perfusion pressure

Real-time perfusion pressure values were measured using a manometer. To create a hydrostatic pressure environment, a perfusion system was designed as shown in Fig. S1A. The system consists of a storage bottle, a peristaltic pump, a culture container, a waste bottle and a pressure manometer (BENETECH, GM-510). The system is completely closed, and the pressure can be maintained at a relatively stable level by adjusting the height of the system.

### Statistical analysis

Values are expressed as the mean ± SD of values obtained from three to six samples. Statistical analysis between two groups was carried out using Student’s t test; comparison among three groups was determined by one-way ANOVA (SPSS16.0). P < 0.05 was considered to be statistically significant.

## Electronic supplementary material

Supplementary Information
