# Supplementary material for: In vitro biomimetic platforms featuring a perfusion system and 3D spheroid culture promote the construction of tissue-engineered corneal endothelial layers
Source: Sci Rep. 2017 Apr 10;7:777. doi: 10.1038/s41598-017-00914-1 (PMC5429708; doi:10.1038/s41598-017-00914-1)
Supplement: Supplementary file 1 — Supplementary Information [file 41598_2017_914_MOESM1_ESM.pdf]

Supplementary Information

In vitro biomimetic platforms featuring a perfusion system and 3D spheroid culture promote the construction of tissue-engineered corneal endothelial layers

Shanyi Li[1], Yuting Han[3], Hao Lei[4], Yingxin Zeng[4, 6], Zekai Cui[1], Qiaolang Zeng[3], Deliang Zhu[1], Ruiling Lian[3], Jun Zhang[4, 5], Zhe Chen[4, 5] & Jiansu Chen[1, 2, 3]

[1] Key Laboratory for Regenerative Medicine, Ministry of Education, Jinan University, Guangzhou 510632, P.R. China

[2]Institute of Ophthalmology, Medical College, Jinan University, Jinan University, Guangzhou 510632, P.R. China

[3]The Department of Ophthalmology, the First Clinical Medical College, Jinan University, Guangzhou 510632, P.R. China

[4]Key Laboratory of Optoelectronic Information and Sensing Technologies of Guangdong Higher Educational Institutes, Jinan University, Guangzhou, 510632, P.R. China

[5]Department of Optoelectronic Engineering, Jinan University, Guangzhou, 510632, P.R. China

[6]Department of Applied Physics, South China Agricultural University, Guangzhou, 510632, P.R. China

Correspondence and requests for materials should be addressed to J.C. (email: chenjiansu2000@163.com) or Z.C. (email: thzhechen@jnu.edu.cn)

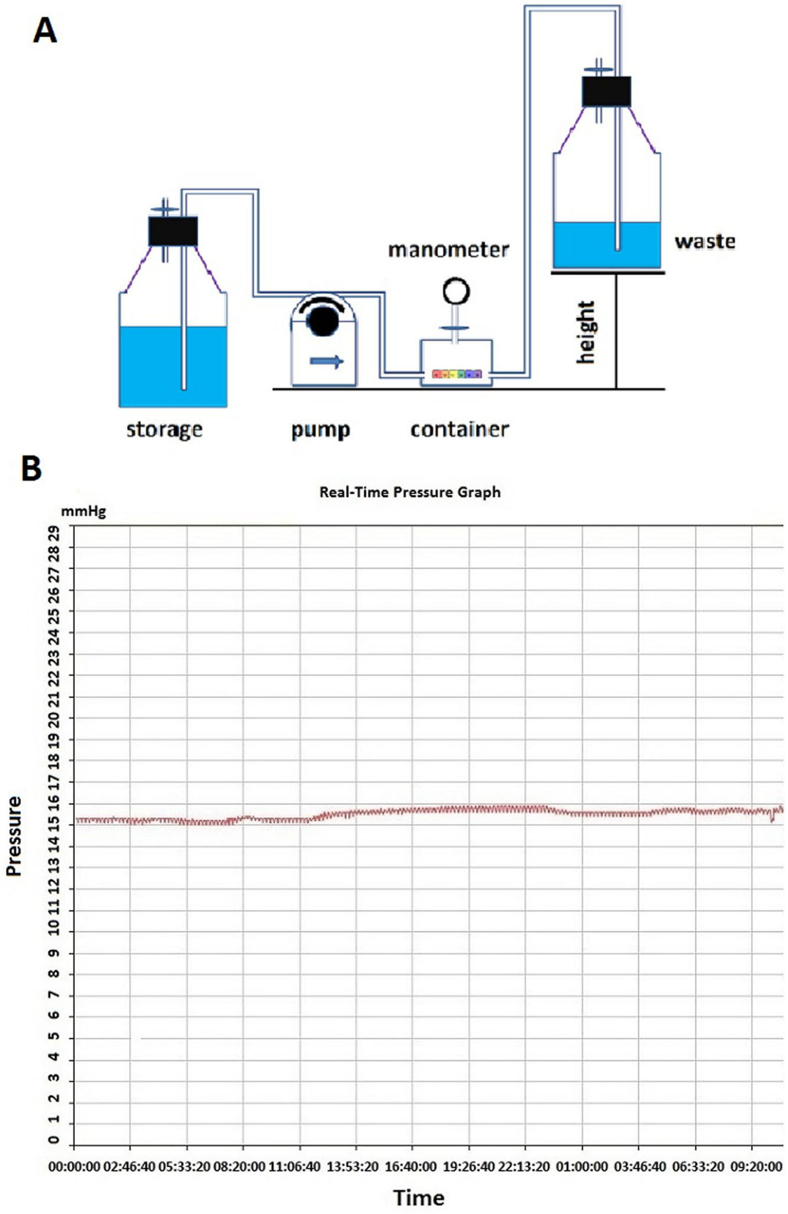

Figure S1. Application of perfusion with controlled pressure. A. Photographic illustration of the perfusion system with controlled pressure. B. Representative real-time pressure maintained at approximately 15 mmHg.

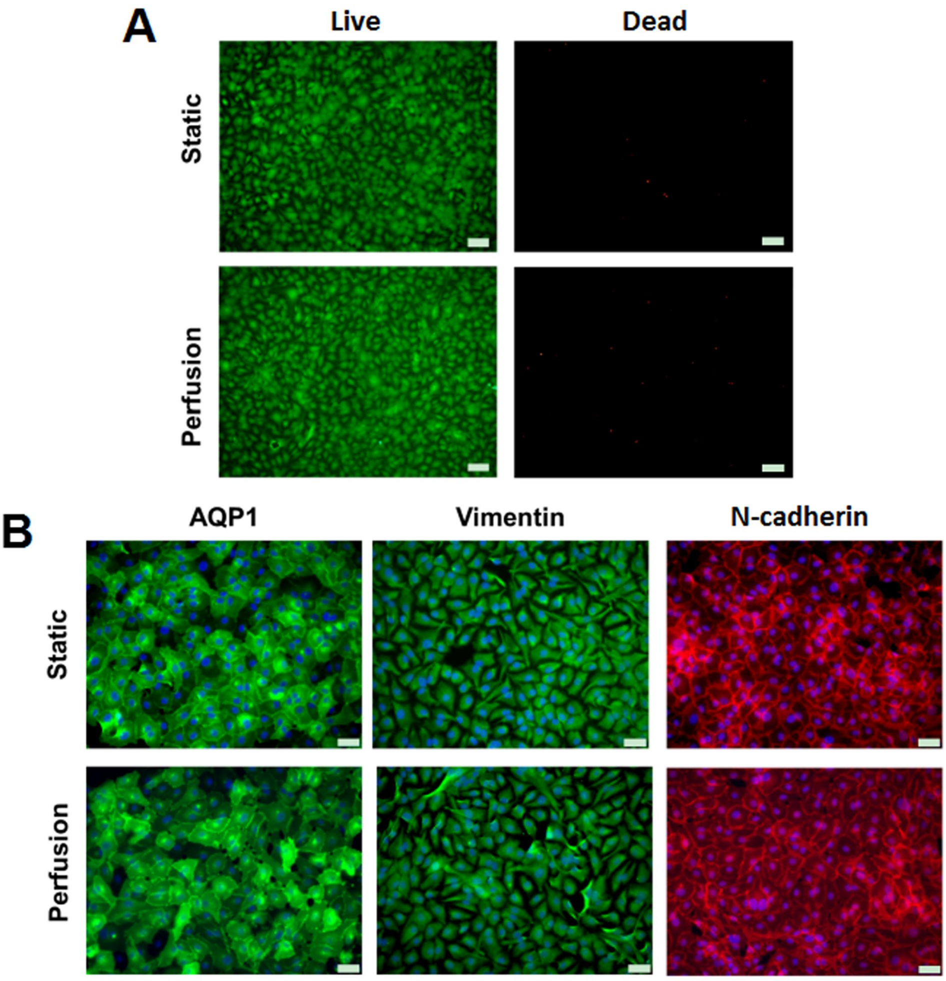

Figure S2. Effects of CECs passaged on 2D culture TCPS after static or perfusion culture on the growth ability and phenotypic expression of CECs. A. CECs passaged on 2D culture TCPS after static or perfusion culture were double-stained with Calcein AM and EthD-III. Scale bar: 100 μm. B. CECs passaged on 2D culture TCPS after static or perfusion culture were stained with anti-AQP1, anti-Vimentin and anti-N-cadherin followed by counterstaining with DAPI. Scale bar: 50 μm.

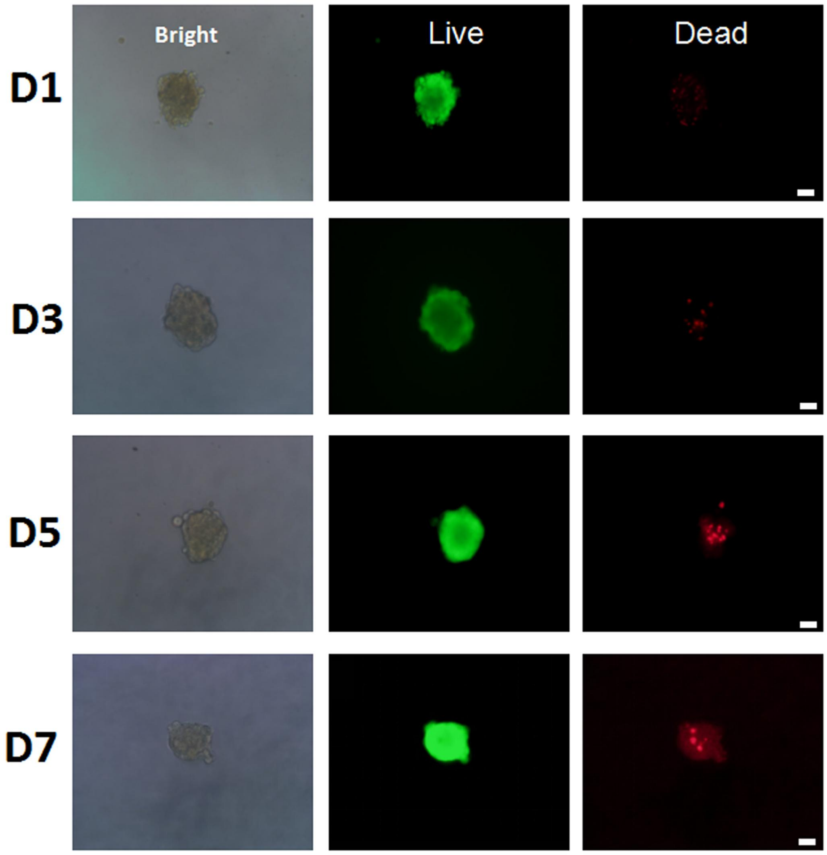

Figure S3. CEC spheroids double-stained with Calcein AM and EthD-III for live/dead cells at D1, D3, D5 and D7. Scale bar: 100 μm.

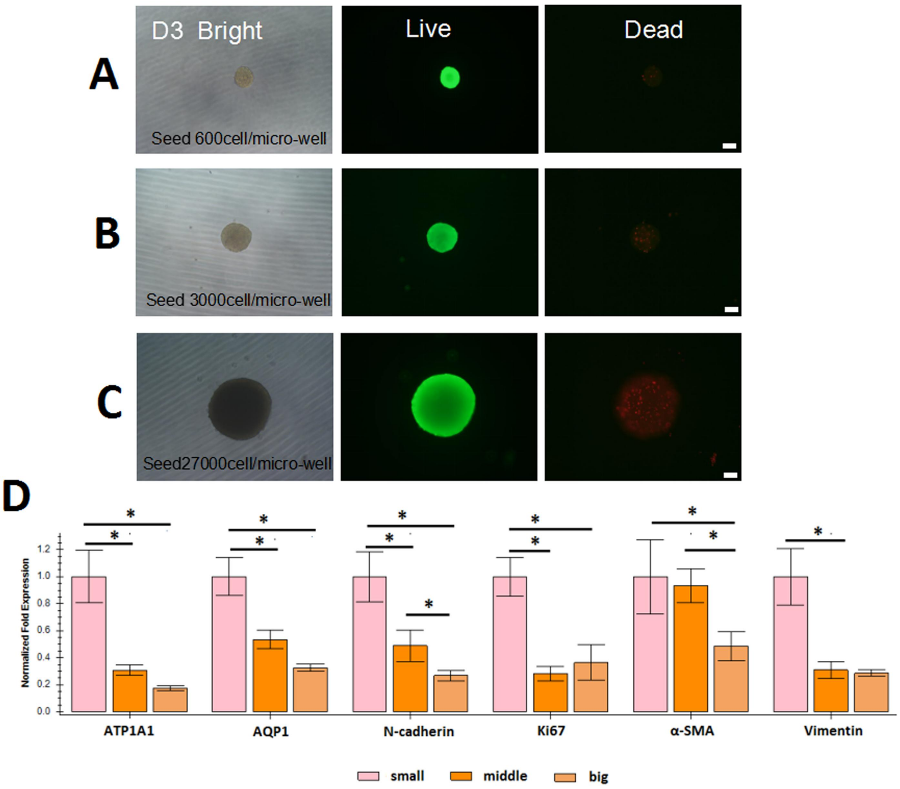

Figure S4. Effects of spheroid diameter on phenotype and gene expression in CEC spheroids. A-C. CEC spheroids were double-stained with Calcein AM and EthD-III to identify live and dead cells at D3. Scale bar: 100 µm. D. QPCR analyses of *ATP1A1, AQP1, N-cadherin, Ki67, α-SMA and Vimentin* expression normalized to *GAPDH* expression. Differences with *P<0.05 were considered statistically significant.

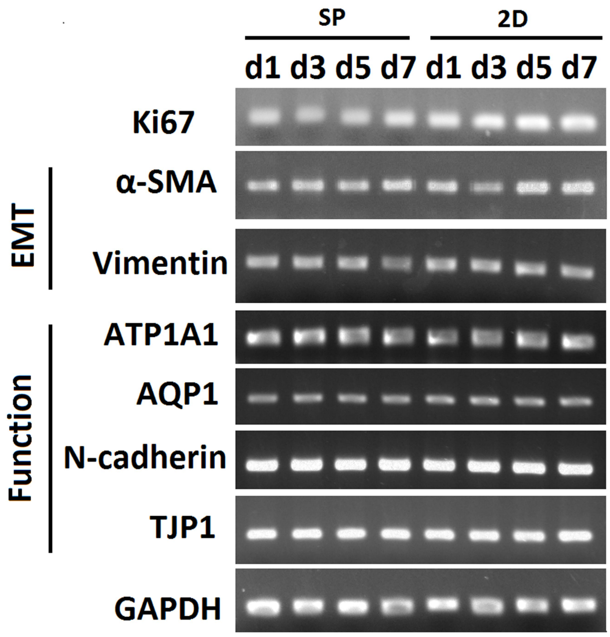

Figure S5. Agarose gel electrophoresis showing the expression of proliferation, EMT and functional markers in CECs.

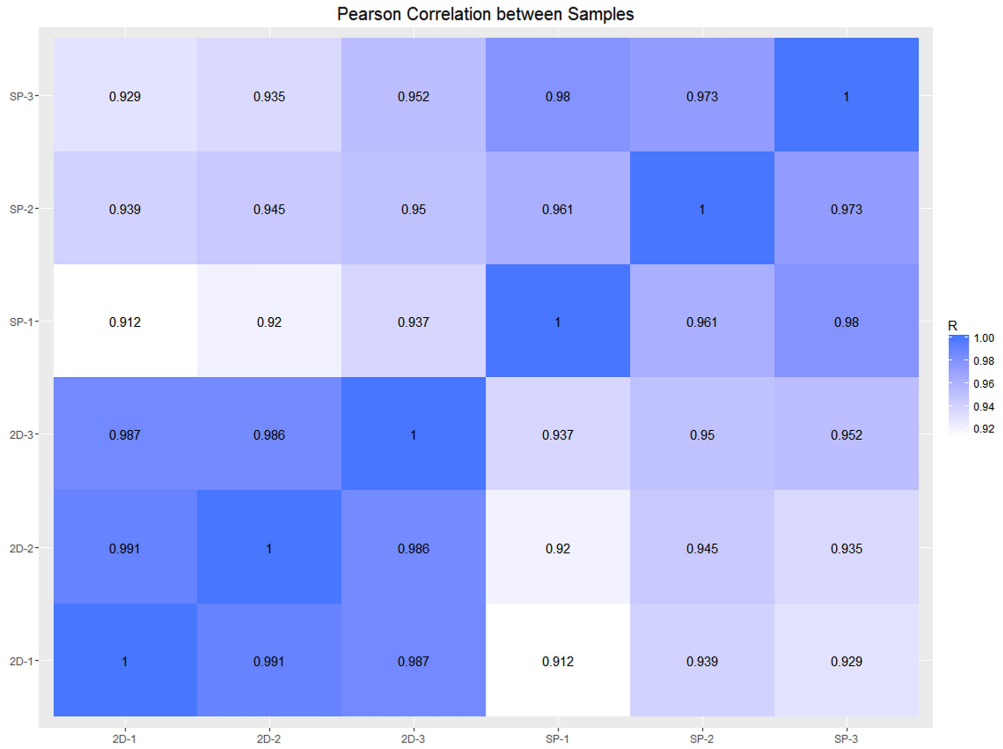

Figure S6. Pearson correlation heatmap of gene expression levels in the 2D and SP cultures.

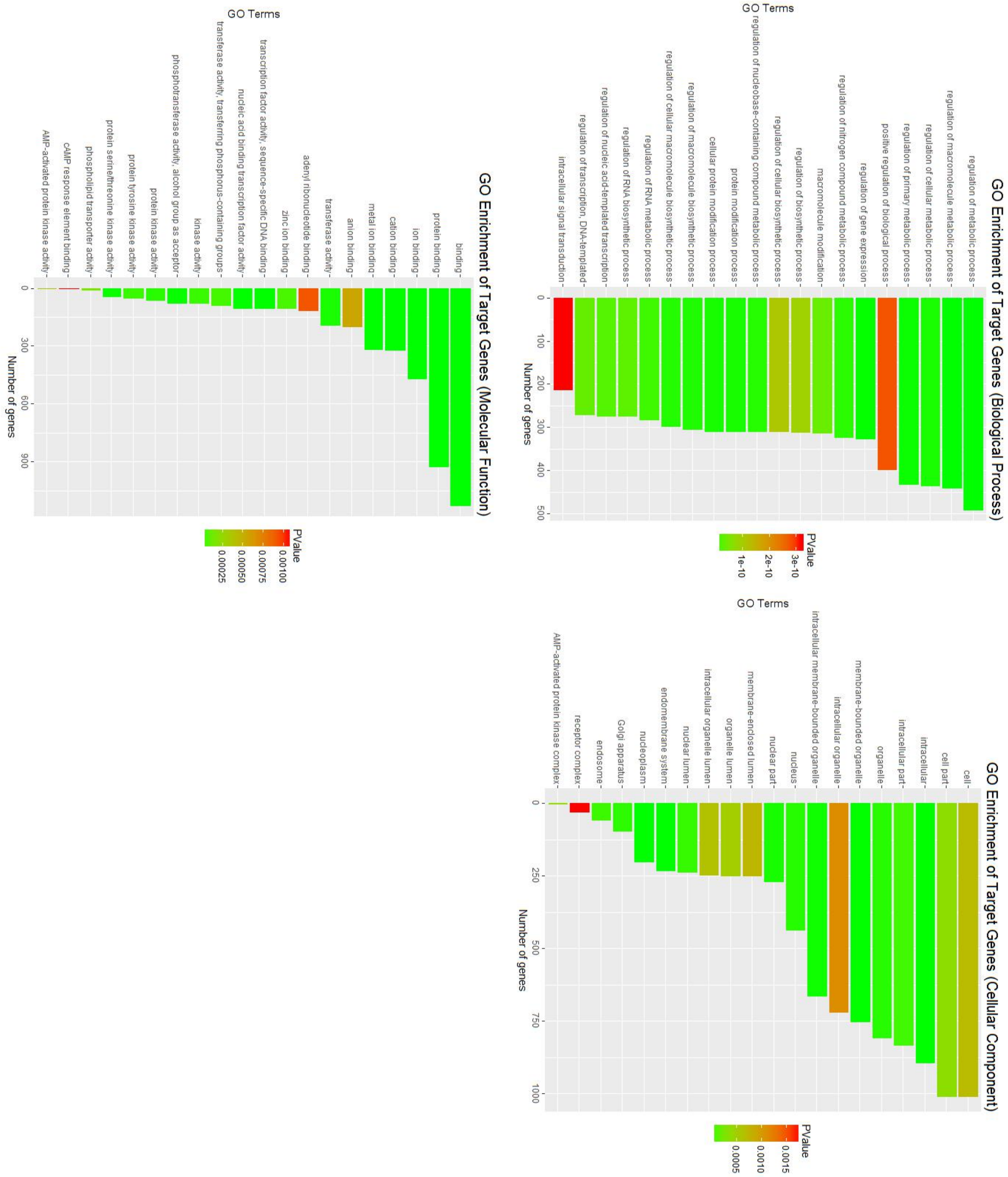

Figure S7. Top 20 GO terms of up-regulated DEGs in 2D and SP cultures (biological process, cellular component and molecular function).

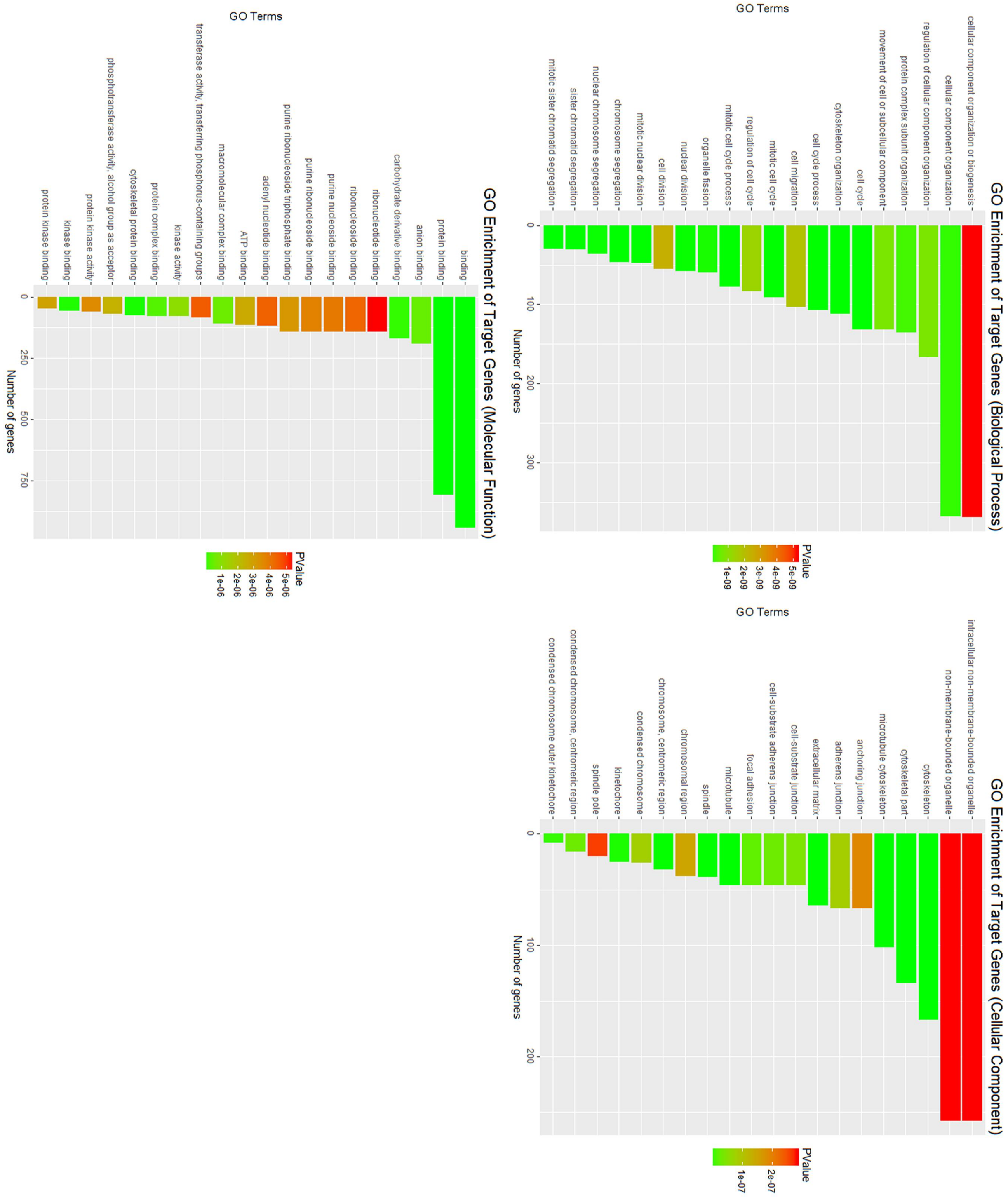

Figure S8. Top 20 GO terms of down-regulated DEGs in 2D and SP cultures (biological process, cellular component and molecular function).

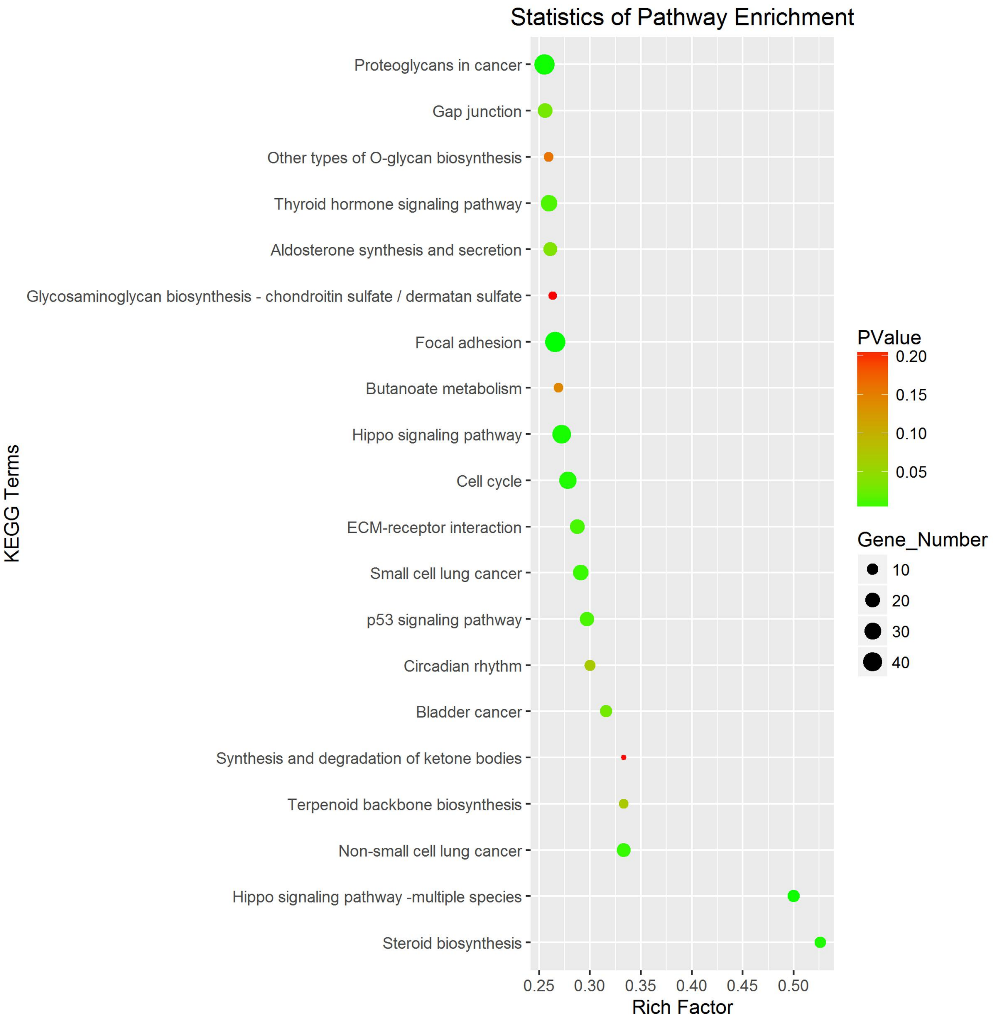

Figure S9. Top 20 statistics of pathway enrichment.

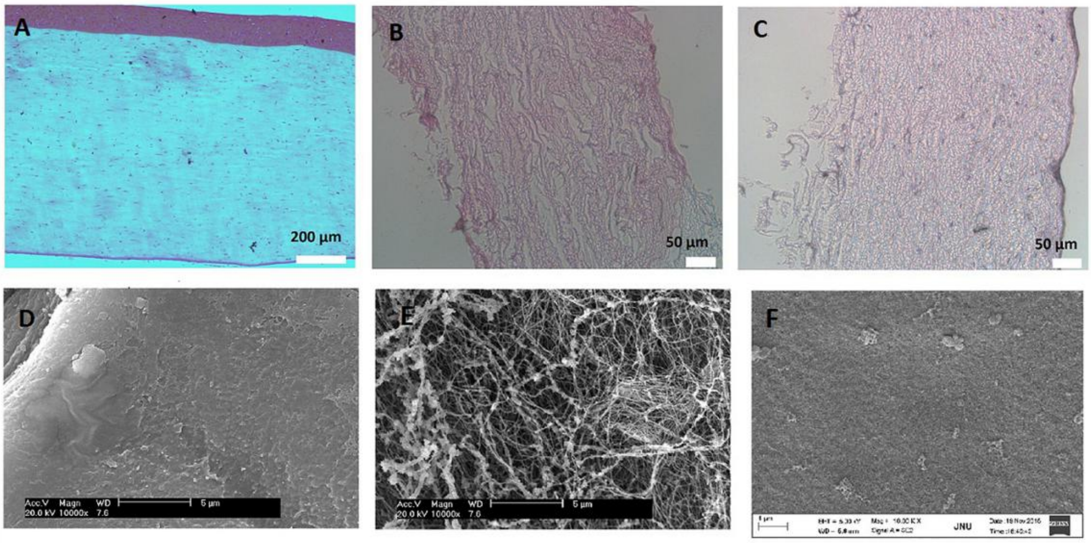

Figure S10. Characteristics of decellularized corneal scaffolds. Representative images of H&E staining of normal (A) and decellularized (B, C) corneas. SEM images of decellularized corneas including Bowman's layer (D), the stromal layer (E) and Descemet's membrane layer (F).

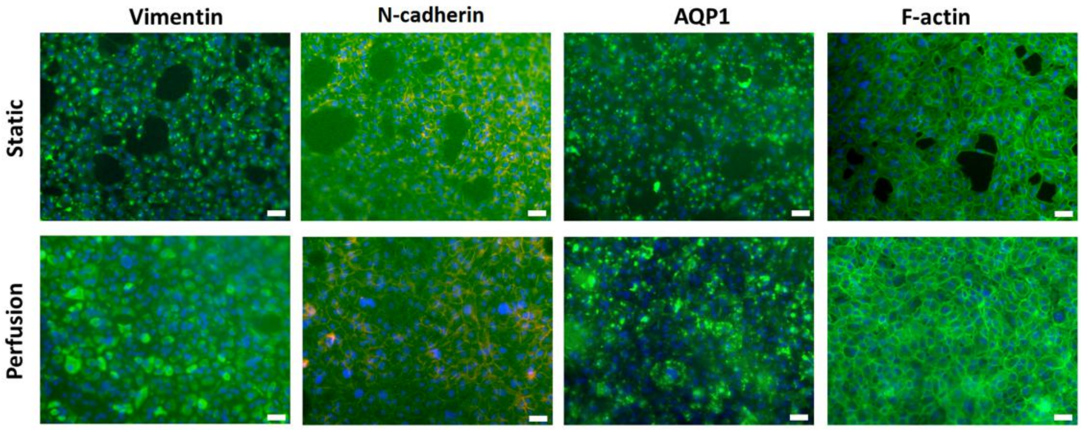

Figure S11. Fluorescence microscopic imaging of CECs cultured on collagen sheets and stained for vimentin, N-cadherin, AQP1 and F-actin. Scale bar: 50 µm.

Gene	Sequence (5' to 3')	Temperature	Length (bp)
Vimentin-F	GGCCCAGATTCAAGAACAGC	60℃	177
Vimentin-R	TTGCGGTTAGCAGCTTCAGA		
TJP1-F	GTCCTGCCCAACTCAACTCA	60℃	165
TJP1-R	CAAGCCTTCTTTGGCAGCAG		
AQP1-F	TCACTGGCCTTTGGGTTGAG	60℃	182
AQP1-R	GAGAGGATGGCAGTGGCAA		
N-cadherin-F	CCTCAAACCAGCCCTACCTG	60℃	223
N-cadherin-R	GCTGTACCGCAGAGAAAGGT		
α-SMA-F	CCAGGGCTGTTTTCCCATCT	60℃	160
α-SMA-R	TCCCAGTTGGTGATGATGCC		
Ki67-F	TGTGGGCCACTTACCACCAA	60℃	128
Ki67-R	CAAAGCCGTAGCCTTTGCTAT		
ATP1A1-F	AATGCGGAAGAGGTTGTCGT	60℃	186
ATP1A1-R	AATGTTCCGTGTCTCCAGGG		
GAPDH-F	GTATGATTCCACCCACGGCA	60℃	129
GAPDH-R	CCAGCATCACCCCACTTGAT		

Table S1. List of primers.
